# Sampling of Protein Conformational Space Using Hybrid Simulations: A Critical Assessment of Recent Methods

**DOI:** 10.3389/fmolb.2022.832847

**Published:** 2022-02-04

**Authors:** Burak T. Kaynak, James M. Krieger, Balint Dudas, Zakaria L. Dahmani, Mauricio G. S. Costa, Erika Balog, Ana Ligia Scott, Pemra Doruker, David Perahia, Ivet Bahar

**Affiliations:** ^1^ Department of Computational and Systems Biology, School of Medicine, University of Pittsburgh, Pittsburgh, PA, United States; ^2^ Laboratoire de Biologie et Pharmacologie Appliquée, Ecole Normale Supérieure Paris-Saclay, Gif-sur-Yvette, France; ^3^ Department of Biophysics and Radiation Biology, Semmelweis University, Budapest, Hungary; ^4^ Programa de Computação Científica, Vice-Presiden̂cia de Educação, Informação e Comunicação, Fundação Oswaldo Cruz, Rio de Janeiro, Brazil; ^5^ Laboratory of Bioinformatics and Computational Biology, Center of Mathematics, Computation and Cognition, Federal University of ABC-UFABC, Santo André, Brazil

**Keywords:** conformational landscape/space, normal mode analysis, molecular simulations, elastic network models, HIV-1 protease, triosephosphate isomerase, 3-phosphoglycerate kinase, HIV-1 reverse transcriptase

## Abstract

Recent years have seen several hybrid simulation methods for exploring the conformational space of proteins and their complexes or assemblies. These methods often combine fast analytical approaches with computationally expensive full atomic molecular dynamics (MD) simulations with the goal of rapidly sampling large and cooperative conformational changes at full atomic resolution. We present here a systematic comparison of the utility and limits of four such hybrid methods that have been introduced in recent years: MD with excited normal modes (MDeNM), collective modes-driven MD (CoMD), and elastic network model (ENM)-based generation, clustering, and relaxation of conformations (ClustENM) as well as its updated version integrated with MD simulations (ClustENMD). We analyzed the predicted conformational spaces using each of these four hybrid methods, applied to four well-studied proteins, triosephosphate isomerase (TIM), 3-phosphoglycerate kinase (PGK), HIV-1 protease (PR) and HIV-1 reverse transcriptase (RT), which provide extensive ensembles of experimental structures for benchmarking and comparing the methods. We show that a rigorous multi-faceted comparison and multiple metrics are necessary to properly assess the differences between conformational ensembles and provide an optimal protocol for achieving good agreement with experimental data. While all four hybrid methods perform well in general, being especially useful as computationally efficient methods that retain atomic resolution, the systematic analysis of the same systems by these four hybrid methods highlights the strengths and limitations of the methods and provides guidance for parameters and protocols to be adopted in future studies.

## Introduction

Under physiological conditions, proteins sample a distribution of conformations while retaining their native fold. Indeed, the dynamic equilibrium of accessible conformations often underlies the regulation of protein function and allosteric mechanisms or their adaptability to bind various ligands or drugs (Haliloglu and Bahar, 2015; Zhang et al., 2020; Wingert et al., 2021). Several studies in the last decade have confirmed the importance of structural dynamics in facilitating, if not driving, the interactions and function of biomolecular systems in the cell (Bahar et al., 2010; Orellana, 2019; Thirumalai et al., 2019; Resende-Lara et al., 2020). In particular, the role of structural dynamics in supporting catalytic activity is a topic of interest (Yon et al., 1998; Bahar et al., 2010; Jiang et al., 2011), with the understanding that enzymes are mechanochemical entities (Yang and Bahar, 2005) and conformational mechanics often complement chemical events by enabling domain or loop movements required for activation.

The determination of 3D coordinates of proteins and their complexes/assemblies has accelerated in recent years thanks to advances in experimental methodologies. Specifically, the developments in cryo-electron microscopy (cryo-EM) and X-ray free-electron laser (FEL) crystallography have revealed multiple snapshots of flexible and complex molecular systems (Branden and Neutze, 2021). In parallel with rapidly growing structural data, theoretical and computational methods that exploit those data toward gaining insights into mechanisms of function have gained importance. While traditional methods, exemplified by molecular dynamics (MD) simulations work as primary tools for studying dynamic events at full atomic details, they still fall short of providing an adequate description of cooperative events at time scales beyond microseconds for multi-domain/multi-subunit systems. On the other hand, analytical methods and coarse-grained (CG) models, exemplified by Normal Mode Analysis (NMA) with elastic network models (ENMs), permit us to solve for the spectrum of modes uniquely accessible to supramolecular systems, providing mathematically exact and physically plausible information on cooperative events, albeit neglecting anharmonicity and atomic details.

Several approaches have been developed aiming to increase the specificity of CG approaches while retaining the high resolution of full atomic simulations. Many ENM-based approaches have focused on the optimization of the basic parameters, spring constants and cutoff distances/functions for inter-residue interactions (Hinsen, 1998; Yang et al., 2009; Kaynak et al., 2018; Kaynak and Doruker, 2019; Koehl et al., 2021), but such studies fall short of providing atomic level information. Instead, another research line, the development of the so-called hybrid methods that combine MD and NMA (using either ENMs or full atomic models) proved to be useful in recent years. These methods have demonstrated two key advantages: 1) an accurate description of cooperative changes in structure, usually described by low frequency normal modes (NMs), and 2) providing atomic details and incorporating local non-linear effects from MD simulations that ‘recalibrate’ these conformational changes (Krieger et al., 2020). Such methods are also beneficial for flexible fitting to cryo-EM maps (Costa et al., 2020) where methods employing either MD or NMA are typically used (Miyashita and Tama, 2018).

In this article, we provide a comparative analysis of such hybrid methods developed for efficient sampling of the conformational space and the possible transitions between functional states. We focus on four methods: ClustENM (Kurkcuoglu et al., 2016), its recent extension ClustENMD (Kaynak et al., 2021), MD with excited NMs (MDeNM) (Costa et al., 2015), and collective MD (CoMD) (Gur et al., 2013). ClustENM produces successive generations of conformers by deforming along low frequency modes, clustering the conformers, and performing energy minimization at full atomic scale. ClustENM conformers have been effectively used in ensemble docking studies for protein-ligand, protein-protein and protein-DNA/RNA pairs (Kurkcuoglu and Doruker, 2016; Can et al., 2017; Kurkcuoglu and Bonvin, 2020), including supramolecules like the ribosome. The recent extension, ClustENMD, uses short MD simulations for the refinement of the generated conformers. The MDeNM method is a multi-replica protocol designed to enhance conformational exploration in a subspace defined by a set of low-frequency NMs, also including the couplings with localized motions occurring within the Cartesian space. In this method, additional atomic velocities are introduced along different linear combinations of NMs. Even though NMs are usually computed in vacuum, they are used as privileged directions in MD simulations with an explicit representation of the surrounding medium. MDeNM has demonstrated its power in conformational sampling in several studies revealing important protein functional movements (Dudas et al., 2020; Dudas et al., 2021a; Fagnen et al., 2020; Fagnen et al., 2021) and has also been successfully used in ensemble docking studies (Dudas et al., 2021b). CoMD provides a combination of ENM-NMA and targeted MD, coupled with energy minimization to adaptively generate a series of conformers.

The metrics for evaluating the performance of these methods deserve attention. For example, while the ability to reproduce crystallographic B-factors has been adopted as a metric in many studies, the comparison of ENM-NMA predictions with the covariance derived from MD simulations were reported to enable a more accurate assessment (Fuglebakk et al., 2013). Here we use the data from both MD and experiments to evaluate the principal components (PCs) of structural changes observed in experiments and predicted by the hybrid methods. The idea, independently introduced in two original studies (Yang et al., 2008; Bakan and Bahar, 2009), is to consider the ensemble of structures resolved for a given protein (e.g. multiple X-ray structures resolved in the presence of different drugs for HIV-1 protease), and examine whether this ‘experimental space’ of conformations matches that predicted computationally. This is a rigorous comparison, unbiased by the selection of conformers used as reference.

**FIGURE 1:**
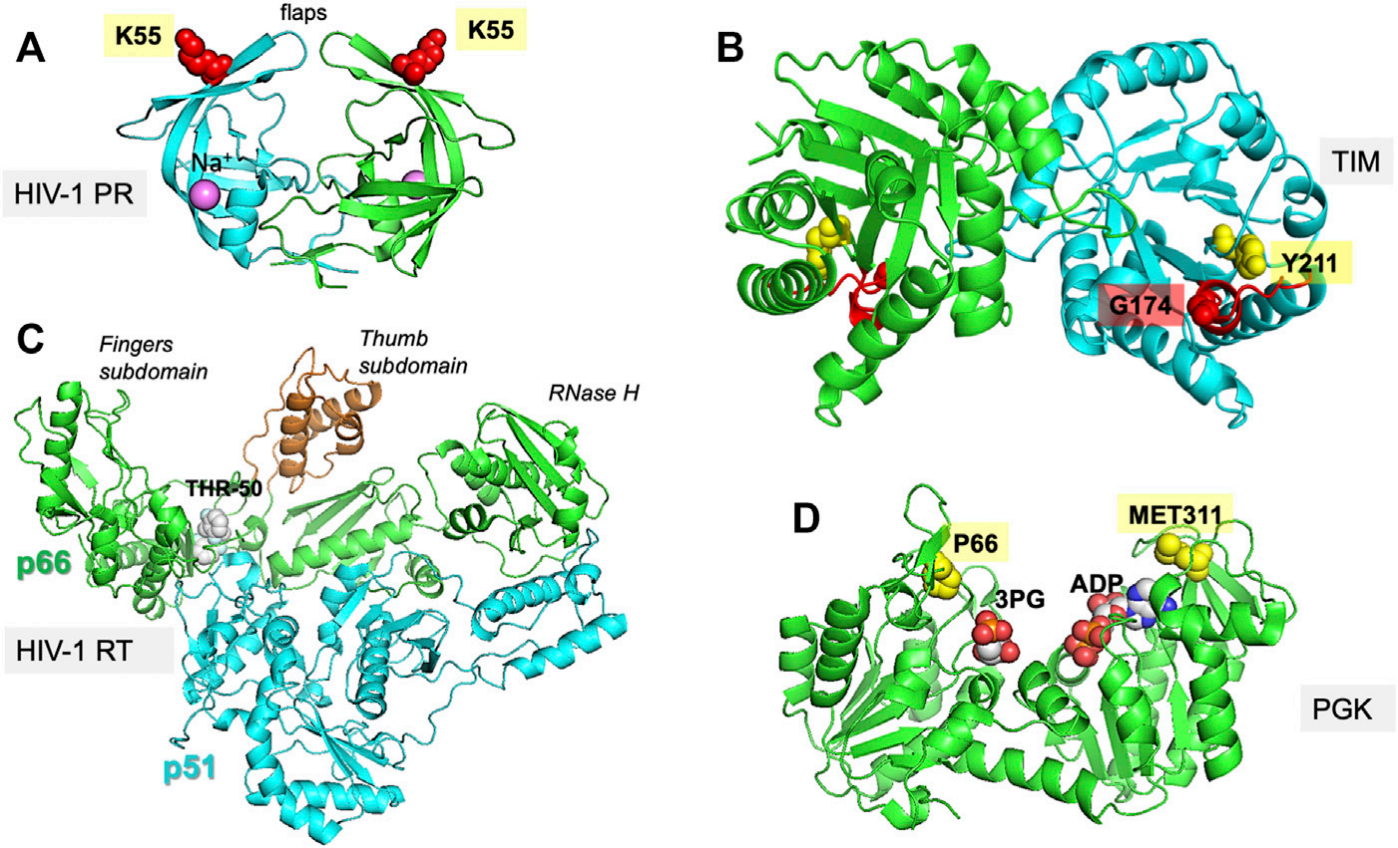
Proteins investigated in the present study. The figure displays the experimentally resolved X-ray structures also used as initial structures for simulations. **(A)** HIV-1 protease (PR) (PDB id: 1tw7) is a wide-open, apo structure. The residue K55 on each subunit of the homodimer isused to probe the opening/closure of the flaps. **(B)** TIM (PDB id:1tcd) is a homodimeric enzyme, for which the catalytic loop is shown in *red* on both subunits of the apo state. The distance between the catalytic loop tip residue G174 and Y211 defines loop opening/closure motion in each subunit. **(C)** HIV-1 RT (PDB id: 2b6a) is a heterodimer composed of p51 and p66 subunits. The current structure is in complex with THR-50. The distance between the fingers and thumb subdomains, both located on the p66 subunit, indicate a transition between closed and open conformations of the region between these two subdomains. **(D)** PGK (PDB id: 2xe7) in the presence of the two substrates, 1,3-bisphosphoglycerate (bPG) and ADP. The distance between P66 and M311, two residues located at the tips of the N- and C-domains near the ligands, probes the opening/closing movement of the enzyme required for its catalytic activity.

We perform our comparative analysis for four well-studied enzymes: triosephosphate isomerase (TIM), 3-phosphoglycerate kinase (PGK), HIV-1 protease (PR), and HIV-1 reverse transcriptase (RT) (Figure 1). Table 1 lists the properties of these enzymes, including the reference structure, the size and oligomeric state of the protein, and the number of experimentally resolved structures used in our comparative analysis along with the corresponding threshold for pairwise sequence identity. Overall, the study serves two major purposes: it provides a rigorous comparison of the performance of the hybrid methods revealing their limitations and advantages, and it helps determine the optimal parameters used in these methods thus permitting us to build fully automated algorithms that can be readily adopted for future applications.

**TABLE 1:** Proteins used as case studies, and corresponding structural data from experiments.

Protein name (acronym)	PDB structure used as References/initial State	Total # of residues^a^ and functional oligomerization state	Number of experimentally resolved structures (and their sequence identity threshold^b^)
HIV-1 protease (PR)	1tw7 Martin et al. (2005); Apo	198 (Homodimer; 99 residues/monomer)	768 (90%)
3-phosphoglycerate kinase (PGK)	2xe7 Zerrad et al. (2011); Complex^c^	413 (Monomer; 416 residues)	35 (90%)
triosephosphate isomerase (TIM)	1tcd Maldonado et al. (1998); Apo	497 (Homodimer; 251 residues per monomer)	57 (50%)
HIV-1 reverse transcriptase (RT)	2b6a Morningstar et al. (2007); Complex with a NNRTI^d^	978 (Heterodimer; 560 residues in p66 subunit, 440 in p51)	365 (90%)

a The number of residues resolved in the reference structure. The actual number is written in parenthesis).

b The percentage in parentheses is the sequence identity threshold after optimal multiple sequence alignment.

c The ternary complex with 3 PG and ADP, in the open state of PGK, was used as reference structure.

d NNRTI: non-nucleoside RT inhibitor.

## Methods

We present below a brief description of the hybrid methods examined here along with the methods used for comparative analysis. Table 2 provides a summary of the parameters and protocols used in each hybrid technique, along with the number of conformers generated for each studied system. Overall, *six ensembles* of structures or conformations are studied: those resolved experimentally, those sampled by MD, and those predicted by four hybrid methods, ClustENM, ClustENMD, MDeNM, and coMD. All methods are applicable to proteins, DNA, and RNA molecules and their complexes.

**TABLE 2:** Parameters, protocols, and outputs of investigated hybrid techniques.

Hybrid method	# Of modes	RMSD^a^ or T (K)^b^	# Of runs	Specification of the methods	# Of conformers generated by the hybrid methods			
					PR	PGK^c^	TIM	RT
CoMD	3	1 Å	9/9/9/10 for PR/PGK/TIM/RT	● 50 cycles of targeted MD guided by ANM	459	450	459	510
				● Monte Carlo-metropolis criteria for selecting new conformers^*e*^				
				● Explicit (TIP3) water and ion				
ClustENM^d^	3	1 Å	3	● 5 generations of ANM sampling	898	900	903	903
				● Energy minimization (EM)				
				● Implicit solvent				
ClustENMD^d^	3	1 Å	3	● 5 generations of ANM sampling	902	903	903	903
				● MD (for heating up the system)				
				● Implicit solvent				
MDeNM	3	2/2/3/3 K for PR/PGK/TIM/RT	1	● Excited MD using atomic NMA	1,560	1,056	415	600
				● 20/8/5/10 re-excitations for PR/PGK/TIM/RT				
				● Explicit (TIP3) water and ion				

a Deformation size or RMSD (Å) is used in coMD, for setting target structures; whereas it is used to generate the conformers in each generation of ClustENM(D) followed by relaxation.

b MDeNM uses an excitation temperature for adding extra velocities to atoms along modes direction. Temperature values separated by slashes correspond to different systems ordered as PR, PGK, TIM, and RT, respectively.

c PGK, ligands were not included in ClustENM, or ClustENMD, but were included for other methods.

d For PR and PGK, a few conformers with high energies have been automatically excluded from the ensemble. The maximum number of conformers from each run is 301.

### ClustENM and ClustENMD

ClustENM (Kurkcuoglu et al., 2016) is a fully automated conformational sampling method composed of multiple generations/cycles consisting each of the following steps: 1) conformer generation by deforming along global NMs calculated using the anisotropic network model (ANM) (Atilgan et al., 2001), 2) clustering of generated conformers, and 3) relaxation of cluster representatives. The cluster representatives will be the parent conformers that are passed onto the next generation of sampling. The ANM modes are updated for each parent conformer in each generation. A series of deformations along the ± directions of a few global modes (3–5) are carried out by targeting a specific root-mean-square deviation (RMSD) for deformation (*Step 1*). Representative conformers selected for computing the next generation of conformers are relaxed by energy minimization (EM) in implicit solvent (*Step 3*).

In the extended version of ClustENM, called ClustENMD (Kaynak et al., 2021), MD simulations using OpenMM (Eastman et al., 2017) are performed for conformational relaxation in *Step 3*. Relaxation can be performed either in implicit solvent (Onufriev et al., 2004) with the Amber99SB force field (Lindorff-Larsen et al., 2010) or explicit solvent. The former is used in this study. In ClustENMD, ANM sampling (*Step 1*) enables deformations along random combinations of global modes with a specified average RMSD from each parent conformer. Both ClustENM and ClustENMD have been implemented in the application programming interface (API) *ProDy* (Bakan et al., 2011; Zhang et al., 2020).

The present analysis allows us to compare two types of relaxation (*Step 3*) using 1) only EM (ClustENM) and 2) EM followed by heating up the system to a desired temperature (here 303.15 K) by MD simulations of about 3 ps at neutral pH (ClustENMD). The same parameters are used for all proteins, namely average RMSD of 1 Å for deformation and five generations of sampling composed each of random combinations of the first three global modes (Table 2).

### Collective Molecular Dynamics

Collective Molecular Dynamics (coMD) simulations were run using scripts generated by an updated version of our previous coMD plugin (Gur et al., 2013; Gur et al., 2015) for VMD (Humphrey et al., 1996) available at https://github.com/prody/coMD. Like the old version, the current coMD plugin uses VMD to prepare the simulation system and interpret the output Tcl script. CoMD uses *ProDy* (Bakan et al., 2011; Zhang et al., 2020) to calculate NMs based on the ANM (Atilgan et al., 2001; Eyal et al., 2015), and NAMD (Phillips et al., 2020) for energy minimization and targeted MD (TMD) (Schlitter et al., 1994; Swift and McCammon, 2008). As previously described (Gur et al., 2013; Gur et al., 2015), ANM modes are selected in a Monte Carlo scheme (ANM-MC) with their probabilities and amplitudes based on their eigenvalues, such that the lowest frequency, or the energetically most favorable, global modes dominate the sampling. coMD can be used to either sample the transition path between two endpoints (with the help of a MC/Metropolis algorithm) or explore the conformational space in the vicinity of a starting conformer (by setting the Metropolis acceptance probability equal to 1). In the current implementation, we adopted the second procedure, given that our goal was to explore the conformational space in the absence of any bias.

Method parameters include the number of modes, maximum deviation per mode, and the total RMSD with respect to the starting conformer at each ANM-MC cycle. A combination of three modes, 0.1 Å deviation, and 1 to 1.5 Å RMSD per cycle was found to give the best compromise between sampling a reasonably large conformational space and avoiding unrealistic deformations such as unfolding. We used a TMD duration of 4 ps to be comparable to other methods. The CHARMM36m (C36m) force field (Huang et al., 2017) was used for all systems. In this study, we used CHARMM-GUI (Jo et al., 2008) systems set up in the other methods (rather than the CoMD VMD plugin) and the Tcl running script was adapted accordingly. This included the liganded complex for PGK as in MDeNM. All other parameters were kept at their default values including 20,000 kcal/mol/Å^2^ for the TMD spring constant.

### MD with Excited Normal Modes

The all-atom NMs required for MDeNM simulations (Costa et al., 2015) were calculated with CHARMM (Brooks et al., 2009) in conjunction with the additive C36m force field (Huang et al. 2017), considering the conformation obtained after an initial short equilibration MD run. The potential energy of the examined structure was minimized till an energy RMS gradient of 10^−5^ kcal/mol/Å was reached. Then, NMs were calculated using the VIBRAN module of CHARMM. The equilibrated solvated structures were considered as starting points for MDeNM simulations. Each MDeNM replica consisted of the conformational exploration along a single linear combination of the selected modes. An RMSD-based filtering was performed before the simulations. Briefly, random linear combinations of the three lowest frequency NMs were followed by 1 Å geometric displacements yielding a deformed structure that must present an RMSD greater than a given threshold (referred to as RMSD threshold) from others previously accepted. If the RMSD of a given generated structure is lower than the threshold, the combination is rejected, and another is generated. This procedure maximizes the variability observed in the excitation directions, therefore covering the defined NM space. These directions are then used to excite the protein kinetically. The excitations are applied periodically each after a given relaxation time during MD simulation, by increasing the atomic velocities along the given excitation direction. As the excitation energy rapidly dissipates, multiple excitations are needed (referred to as the number of excitations). Each excitation increases the temperature of the system by a given amount (called the excitation temperature). Each excitation step is followed by a relaxation time. In line with other studies, we found that excitation energies of 2–3 K coupled with relaxation times ranging from 4 to 8 ps (Costa et al., 2015; Floquet et al., 2015; Dudas et al., 2020, 2021a, 2021b) are broadly applicable. The number of cycles varies by system (Table 2). The intermediate conformers at the end of each excitation-relaxation cycle are collected to define the MDeNM ensembles. The other parameters are the same as those described below for MD simulations.

### MD simulations

We carried out MD simulations in NAMD for comparison of the conformers sampled in MD trajectories with those generated by the hybrid techniques. For each protein, we carried out three independent runs of 200 ns each, explicit solvent at 303.15 K. The systems were prepared using CHARMM-GUI (Jo et al., 2008) and default parameters were used for MD simulations. A set of 6,000 snapshots have been collected at 100 ps intervals for each protein (2,000/run × 3 runs), forming the MD ensembles.

### Comparison Between Computationally Predicted and Experimentally Resolved Ensembles

To compare the simulation outputs with the available experimental data, ensembles of experimentally resolved structures were compiled and subjected to PCA for each of the four proteins studied here (Table 1) using methods developed within the *ProDy* API (Bakan et al., 2011; Bakan et al., 2014; Zhang et al., 2019; Zhang et al., 2020; Zhang et al., 2021). The structures were projected onto the reduced space spanned by the first two PCs defined by the *e*xperimental dataset (*e*PC1 and *e*PC2) for each studied protein, using *ProDy* and visualized by Matplotlib (Hunter, 2007). The corresponding conformers generated by computational methods were also projected onto that subspace, thus allowing for comparison of their distribution in the backdrop of experimentally observed conformational space. Continuous population density plots of conformers were generated for each ensemble using kernel density estimate (KDE) plots from the Seaborn Python package (Waskom, 2021). We used *ProDy* for calculating the RMSDs and distance measures representing the departure from the different functional states of the proteins. The variances of computationally predicted conformers along *e*PC1–*e*PC10 were determined by projecting them onto these *e*PCs and evaluating their standard deviation from the mean.

We also determined the simulated PCs (sPCs) for each ensemble of conformations generated by MD, ClustENM, ClustENMD, MDeNM, and coMD, to determine the dominant modes of conformational changes predicted by the simulations or hybrid methods. Finally, to quantify the extent of similarity between the major structural variations observed in experiments and those predicted by simulations, we evaluated the correlation cosines between experimentally sampled top four PCs (*e*PC1-4) and those sampled in simulations (*s*PC1-4). Likewise, similar correlation cosines were evaluated for pairs of outputs from different computational methods. The results are presented in heat maps (6 × 6 super-matrices), the super-elements of which (4 × 4 blocks) describe the pairwise correlation cosines, or the so-called *overlaps* between pairs of PCs from different methods.

Furthermore, we used the root-weighted square inner product (RWSIP) (Carnevale et al., 2007) as another metric to assess the overall consistency between the spectrum of structural changes observed in simulations and those from experiments, defined as

$$RWSIP={\left[\frac{\sum_{i=1}^{N}\sum_{j=1}^{N}\lambda_{i}^{u}\lambda_{j}^{v}{\left(u_{i}\cdot v_{j}\right)}^{2}}{\sum_{i=1}^{N}\lambda_{i}^{u}\lambda_{i}^{v}}\right]}^{1/2}$$ (1)

Here $\lambda_{i}^{u}$ and $\lambda_{j}^{v}$ are the eigenvalues of the covariance matrices corresponding to their respective PCs, $u_{i}$ and $v_{j}$ (*s*PCs and *e*PCs). *N* is the number of the PCs (*N* = 4 in our analysis). RWSIP takes into account the relative contribution (eigenvalue) of each PC, thereby giving larger weights to the more collective modes.

## Results and Discussion

### HIV-1 Protease (PR)

*HIV-1 protease* (PR) is a homodimeric enzyme consisting of two symmetrically positioned monomers of 99 amino acids each, with the substrate-binding site at the interface of the monomers. The access to this site is mediated by two opposing β hairpins known as flaps (Figure 1A). Several studies have pointed to the significance of the coupled movements of the two PR monomers in relation to catalytic activity. Three regions recognized to be functionally important in each monomer are: 1) the N- and C-terminal residues 1–4 and 95–99, essential to dimer assembly; 2) the central region (residues 10–32 and 63–85) of each monomer containing the catalytic site, and also involved in dimerization; and 3) the highly flexible glycine–rich flaps exposed to solvent (residues 33–62) (Scott and Schiffer, 2000; Henzler-Wildman et al., 2007; Palese, 2017). The opening/closing of the flaps and the twist motion of the two monomers with respect to each other serve as collective motions that support the enzymatic function, coupled to the catalytic dyad dynamics (Batista et al., 2011; Badaya and Sasidhar, 2020).

#### Conformational Variability From Experiments and Computations

**FIGURE 2:**
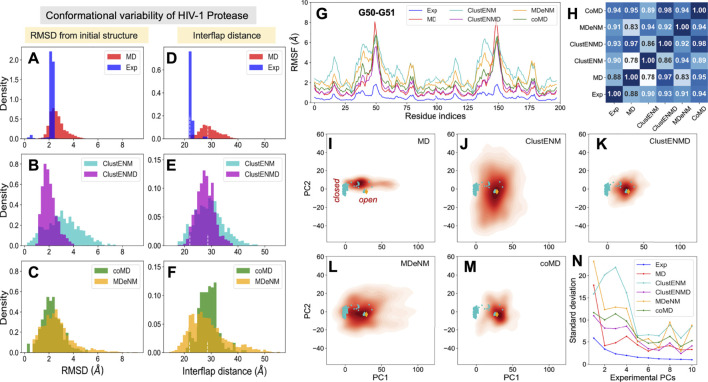
Comparison of computational and experimental ensembles of HIV-1 PR. Histograms: **(A–C)** RMSDs with respect to the starting open structure (PDB id: 1tw7). **(D–F)** Distances between α−carbons of K55 on different subunits that monitors the opening/closure of flaps. *Dashed lines* indicate the distances of the initial open structure and a closed crystal structure (PDB id: 1bve). The distribution for each ensemble, namely experimental (*blue*), MD (*red*), ClustENM (*cyan*), ClustENMD (*magenta*), coMD (*green*), and MDeNM (*orange*), is shown on the shared x-axis. **(G)** RMSFs as a function of residue index for each ensemble, **(H)** Pearson correlation coefficients between all pairs of RMSF profiles. **(I–M**) Population distributions of ensembles of conformers generated by simulations, projected onto the subspace spanned by experimental *e*PC1 and *e*PC2, shown for **(I)** MD simulations, **(J)** ClustENM, **(K)** ClustENMD, **(L)** MDeNM, and **(M)** coMD. *Cyan circles* represent the experimental structures, and the *orange diamond* is the initial structure (PDB id: 1tw7). **(N)** Standard deviations of the conformers projected along the experimental PCs.

PR is one of the most thoroughly studied enzymes as a target for HIV-1 drug development, with over 750 structures resolved to date in different forms, in the presence of different ligands/drugs. Figure 2A provides information on the conformational variability of the 768 PDB structures used here as the experimental dataset. The *blue* histogram in panel **A** displays the RMSDs (based on C^α^ atoms) of these structures from the wide-open form [PDB id: 1tw7 (Martin et al., 2005)] used as reference, showing that the crystallographic structures are rather narrowly distributed (within 2.3 Å RMSD). The ensemble of conformations sampled during MD simulations (*red histogram*) exhibits a broader distribution (up to 4.2 Å), comparable to those generated by ClustENMD and CoMD but narrower than those generated by ClustENM and MDeNM (Figures 2B,C).

As mentioned above, flap opening is required for the substrate to access the active site, and its closure for proteolytic cleavage to occur. The degree of opening of the flaps can be evaluated through the distance between the C^α^ atom of residue K55 in the two monomers (Figure 1A). Figures 2D–F shows distributions of this distance obtained for each ensemble. The *white vertical dashed lines* indicate the distances corresponding to the open and closed states of the flap, represented by the reference structure (open) and 1bve (Yamazaki et al., 1996) (closed). Most of the experimentally resolved structures are closed conformers in the presence of a bound ligand. The PR stability is increased in the bound form such that it is better protected against self-cleavage and its crystallization is facilitated. Again, ClustENM and MDeNM sample significantly more open conformations compared to MD, while ClustENMD and coMD yield inter-flap distances comparable to those sampled in MD.

The conformers generated by the hybrid methods encompass both the open and closed states of the enzyme. The distributions of conformers are continuous and unimodal in MD, ClustENM and ClustENMD, while coMD and MDeNM yield distinct peaks separating the closed form (Figures 2E,F). Notably, the open state is more populated than the closed in all simulations, in contrast to experimental structures. This could be attributed to the fact that simulations were carried out using a wide-open unliganded, drug-resistant mutant (PDB id: 1tw7) as the initial conformation. Note that ligand binding usually favors closed conformers; whereas the unliganded PR preferentially adopts open conformers predisposed to ligand-binding.

#### Residue Fluctuation Profiles

While the RMSDs and inter-flap distances point to broadly distributed ensembles of conformers predicted by the hybrid methods (especially ClustENM and MDeNM), it is of interest to assess whether the residue fluctuation profiles exhibited by those ensembles differ from those observed in experiments and in MD simulations. Figure 2G displays the root-mean-square-fluctuations (RMSFs) of C^α^ atoms from their average positions in each ensemble. As expected, all computations yield higher RMSFs than those observed in the X-ray crystallographic ensemble (reflecting the restricted residue mobilities in the crystals), and ClustENM and MDeNM ensembles exhibit the highest RMSFs. However, the RMSF shapes (profiles as a function of residue index) deduced from experiments and simulations are very similar, as quantified by the pairwise Pearson correlation coefficients (Figure 2H). All four hybrid methods exhibit correlation coefficients higher than or equal to 0.9 with experimental data (and among themselves), showing that a robust pattern of residue fluctuations, albeit the increased amplitudes, is captured by all ensembles. We note that MD simulations yield sharp peaks around G50-G51. This region corresponds to the tips of the flaps, indicating that MD may overestimate these local motions, relative to others that move concertedly. However, the correlation between MD and experiments is still strong (0.88), and those with ClustENMD and coMD are remarkably high (≥0.95).

#### Conformational Landscape

The above analyses compared the conformational diversity and residue flexibilities of the ensembles. Next, we proceed to a closer inspection of the conformational space explored by each method. To this aim, we first determined the subspace spanned by the principal components *e*PC1 and *e*PC2 obtained from the PCA of known structures. The known structures projected onto this subspace are displayed in Figures 2I–M by the *cyan dots*, each dot representing a PDB structure. The cluster of dots on the left refers to closed structures, and the reference (open) structure is displayed by the *orange diamond*. The origin of the plot represents the “average” structure, which lies in the region occupied by the closed structures due to the predominance of closed structures in the experimental ensemble. Next, we evaluated the distribution of conformers for each computationally generated ensemble, projected onto the same subspace. These distributions are displayed by contour plots (*orange-to-red shades*) in Figures 2I–M. The shading/levels get darker as the population density increases. The color-coded contour plots exhibit features consistent with the RMSDs in panels **A-C**.

The subspace spanned by the experimentally derived *e*PC1 and *e*PC2 provides only a partial view of the spread of conformers generated by computations, as some of the conformers may be broadly dispersed along other *e*PCs. As a measure of the variance of computationally generated conformers along additional *e*PCs, we evaluated the standard deviation of the distribution of each computed ensemble of conformers projected along the first 10 *e*PCs. The results are presented in Figure 2N. Highest variations are observed along *e*PC1 followed by either *e*PC3 (ClustENM, MDeNM and coMD) or *e*PC4 (ClustENMD and MD) for all computed ensembles, while the variations along higher modes drop sharply in all cases. These results suggest that the first four *e*PCs are sufficient to describe to a good approximation the diversity of experimentally resolved structures as well as a divergence in the computed conformers (e.g., by ClustENM) with respect to experiments.

#### Comparison of Global Modes/Principal Directions of Motion

Given the important contribution of the top four PCs, we carried out a detailed comparison of the overlap between *e*PC1-4 from experiments, and *s*PC1-4 from each simulation (MD and four hybrid methods). The heatmap in Supplementary Figure S1 provides information on the overlap between these six sets of PCs, organized in a super-matrix of 6 × 6 blocks. Each block (4 × 4 matrix) describes the correlation cosine between the top four PCs corresponding to a pair of ensembles. This way, one can trace back the similarities in the observed conformational heterogeneities to similarities between top-ranking PCs. The *bottom row* shows that *e*PC1 strongly correlates with *s*PC1 from MD and MDeNM (with respective correlation cosines of 0.84 and 0.76), and with *s*PC2 from coMD (0.73). As to the *e*PC2, we note its high correlations with ClustENM *s*PC2 (0.71) and MDeNM *s*PC3 (0.73). Likewise, the second *block-row* from bottom shows the moderate correlations between MD and hybrid methods, and the top four block-rows show strong correlations between the hybrid methods. Thus, even though the order of the PCs may differ, the six ensembles of structures/conformations exhibit equivalent pairs of PCs which predominantly define the observed distributions of conformers. We have furthermore evaluated the RWSIP values [Eq. (1)] as an additional metric for comparison. All hybrid methods as well as MD simulations yield satisfactory correlation (varying as 0.62 ≤ RWSIP ≤0.83) with the experimental ensemble (Table 3) in line with a previous study showing close correspondence between NMs and ePCs for this system (Yang et al., 2008).

**TABLE 3:** Comparative assessment of the performance of hybrid methods.

Protein	ClustENM	ClustENMD	MDeNM	coMD	MD	Experimental
**Closest distance of approach (Å) (** Figure 2 **,** Figure 4 **,** Figure 6 **,** **and** Figure 8 **; panels D–F)** ^**a**^						
PR (K55-K55)_	**17.7**	**17.6**	**14.3**	**20.8**	**21.0**	21.5
TIM (G174-Y211)	**13.8**	**12.0**	14.9	**13.4**	**13.4**	12.9
PGK (P66-M311)	**17.4**	**20.4**	**21.5**	**18.1**	24.9	23.6
RT (thumb-fingers)	28.8	29.9	**26.8**	33.4	39.2	26.9
**Minimum RMSD (Å) from the closed structure** ^**b**^						
Initial structure^**c**^	Attained in simulations, starting from the open state					Initial RMSD (exp)^**d**^
PR (1tw7)	2.2	**1.7**	**1.8**	2.3	**<2**	2.7 (1bve)
PGK (2xe7)	**1.4**	**1.7**	2.9	2.5	3.2	3.6 (2wzb)
RT (2b6a)	3.5	3.8	4.3	3.8	4.7	5.2 (3kli)
**RWSIP** ^**e**^ **with respect to experimental PCs**						
PR	0.62	0.75	0.77	0.72	**0.83**	1.00
TIM	0.54	**0.58**	0.55	0.54	0.52	1.00
PGK	0.87	**0.89**	0.78	0.78	0.74	1.00
RT	**0.72**	0.70	0.56	0.56	0.43	1.00
Average	0.69	**0.73**	0.66	0.64	0.63	—

a Those entries with Δd < 1 Å [where Δd = d (comp)—d (exp)] are shown in boldface.

b Minimum RMSD, of each ensemble with respect to the closed structure reflects the extent of approach from open-to-closed state (those values below 2.0 Å are highlighted in bold). TIM is not included as experimental structures do not show a global transition between open and closed states but just loop motions (RMSD within ∼ 1 Å RMSD).

c PDB id for open structure is in brackets.

d PDB id for closed structure is in brackets.

e The highest (best) RWSIP value observed for each protein is highlighted in bold.

**FIGURE 3:**
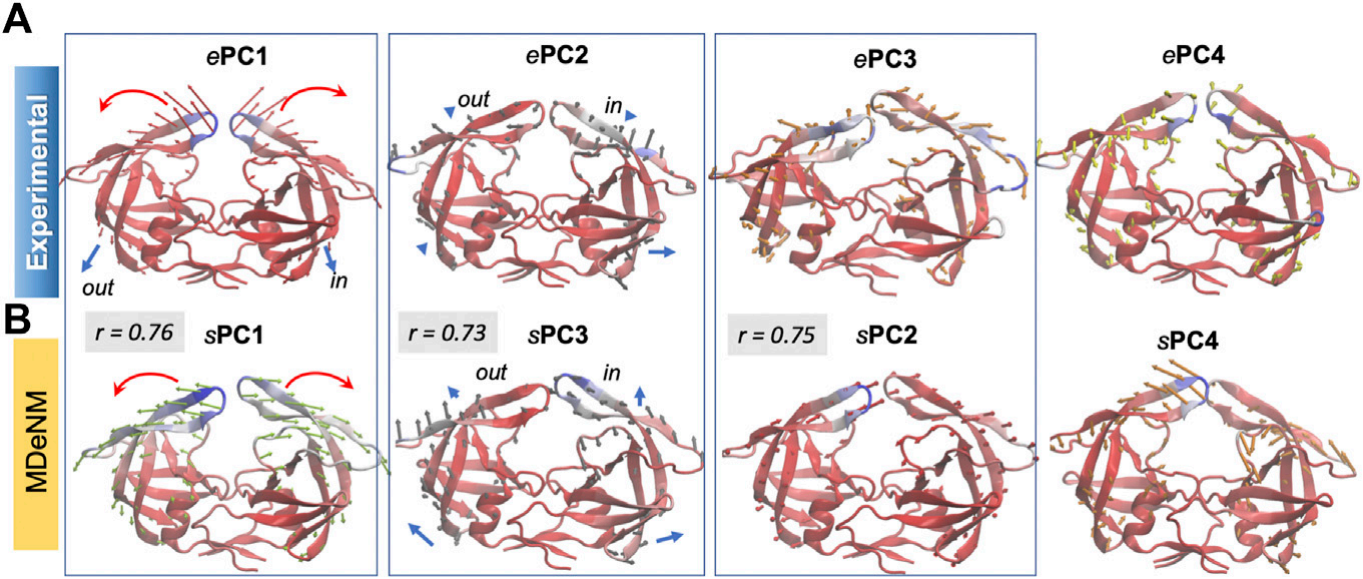
First four principal modes for HIV-1 PR ensembles. The **(A,B)** are based on the PCAs of the experimental and MDeNM ensembles, respectively. Those PCs that exhibit high (>0.70) correlations are enclosed in boxes. See the corresponding movies in Supplementary Movie S1.

The structural variations described by the first four *e*PCs are schematically described by the color-coded ribbon diagrams in the upper panel of Figure 3. The lower panel displays the first four *s*PCs generated by MDeNM. The *s*PCs are reordered to highlight (*by boxes*) the *s*PCs equivalent to the *e*PCs. Notably, *e*PC3 also shows a high correlation (0.75) with MDeNM *s*PC2 and exhibits a bending of the whole structure where the flaps move together. These functional movements along MDeNM *s*PC1-3 can be viewed in Supplementary Movie S1. Earlier studies have shown that the first two collective modes of PR describe internal movements allowing for substrate binding and catalysis (Scott and Schiffer, 2000; Batista et al., 2011; Palese, 2017). We see here that the first two modes, *e*PC1/*s*PC1 and *e*PC2/*s*PC3 capture these flap opening/closure and twisting events, as well as the coupled twisting and bending of the monomers; and *s*PC3 accounts for inter-subunit counter-rotation.

### Triosephosphate Isomerase

*TIM* is a homodimeric enzyme, each subunit adopting a TIM barrel fold (Figure 1B). It plays a key role in the glycolytic pathway catalyzing the interconversion between two triose phosphate sugars, dihydroxyacetone phosphate and D-glyceraldehyde 3-phosphate. The active sites are located at the C-terminal end of each β-barrel. A crucial feature of TIM functional dynamics is the catalytic loop opening/closure on each subunit. Catalysis takes place when the loop is closed protecting the active site from solvent exposure. Loop closure is not ligand-gated, i.e., it takes place in the apo state as well (Williams and McDermott, 1995; Cansu and Doruker, 2008).

#### Conformational Variability From Experiments and Computations

**FIGURE 4:**
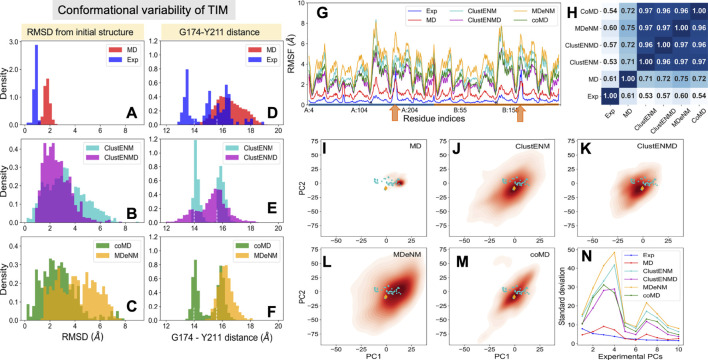
Comparison of the ensembles of conformers experimentally observed and computationally generated for TIM. The panels are in the same format as Figure 2. **(A–C)** RMSD histograms with respect to the starting apo structure (PDB id: 1tcd). **(D–F)** Distances between α-carbons of G174 and Y211 that monitors the opening-closing of the catalytic loop. *Dashed lines* indicate the loop distances in the different subunits of starting dimeric structure. **(G)** RMSFs with respect to the initial structure for each ensemble. *Orange arrows* indicate the catalytic loop position in each subunit. **(H)** Pearson correlation coefficients between pairs of RMSF profiles. **(I–M)** Population distributions of computed ensembles (labeled in each panel) projected onto subspace spanned by *e*PC1 and *e*PC2. **(N**) Standard deviations of the conformers projected along ePCs.

In contrast to the other examined proteins, homologous TIM structures with ≥90% sequence identity to the Trypanosoma cruzi structure used here (PDB id: 1tcd) yielded a small set that closely retained the same structure. To increase structural diversity, we have relaxed the threshold sequence identity to 50%, which led to an ensemble of 57 resolved structures for TIM homologs. The *blue* histogram in Figure 4A displays their distributions (RMSDs) with respect to the starting conformer [PDB id: 1tcd (Maldonado et al., 1998); Table 1]. MD simulations also exhibited a narrowly distributed RMSD histogram (panel A; *red histogram*) while the hybrid methods (panels B–C; labeled) yielded substantially higher RMSDs, pointing to the ability of these methods to sample a broader conformational space, as already seen for HIV-1 PR. Yet, given the high stability of the TIM fold, the relatively lower RMSDs predicted by ClustENMD and coMD could be more realistic.

The catalytic loop motion of TIM can be monitored by the distance change between the C^α^ atoms of G174 (tip residue of loop 6) and Y211 (a relatively immobile residue on the barrel used as reference) (Figure 1B). Our previous MD simulations (Kurkcuoglu and Doruker, 2013; Kurkcuoglu et al., 2015) indicated multiple opening/closing events between this pair of residues. Figures 4D–F shows the distributions of this distance for both subunits. Experiments show a multimodal distribution varying over a broad range (13–19 Å), with the lower values corresponding to the closed loop. In the reference crystal structure (PDB id: 1tcd), the distances are 14.0 and 15.6 Å for A and B monomers, respectively, shown by *white vertical dashed lines* in each panel. ClustENM and coMD exhibit bimodal distributions with peaks localized around these values. Relaxation by heating up in ClustENMD enhances loop flexibility leading to a broader distribution (panel **E**; *magenta*). MD and MDeNM, on the other hand favor the open state only, missing the closed state of the loop observed in experiments and other hybrid methods.

#### Residue Fluctuation Profiles

The RMSFs and their Pearson correlation coefficients are presented in the respective panels G and H of Figure 4. Consistent with RMSDs, the conformers generated by hybrid methods, and especially MDeNM and ClustENM display higher RMSFs compared to those observed in MD simulations and experiments. The two *orange arrows* along the abscissa in Figure 4G indicate the location of the catalytic loops. Thes showed the highest conformational diversity in experiments (*blue curve*). All hybrid methods display similar profiles, but their correlations with experimental ensembles, which vary in the range 0.53–0.60, are much lower than that (0.90–0.94) observed for HIV-1 PR. Their correlations with MD vary from 0.71 to 0.75. The hybrid methods consistently show very high correlations among themselves (>0.95), suggesting that they robustly sample similar motions, beyond those observed in X-ray crystals as will be further elaborated below.

#### Conformational Landscape

Figures 4I–M display the loci of the 57 experimentally resolved structures (*cyan dots*) in the reduced space spanned by *e*PC1 and *e*PC2, and the distribution of computationally predicted ensembles are displayed by the color-coded KDE plots. The origin of each plot corresponds to the mean experimental structure, which lies in proximity to the reference structure (PDB id: 1tcd, *orange diamond*). The experimental structures (*cyan dots*) and MD simulations cover a limited portion of this subspace compared to the hybrid methods with a clear shift of the MD distribution relative to the experimental one in line with the RMSDs and loop distances. Figure 4N describes the distributions of the computationally generated conformers along the first 10 *e*PCs. Higher variations in conformations are observed along the *s*PCs 3 and 4 for all hybrid methods. Therefore, the first two experimental PCs, *e*PC1 and *e*PC2, are not sufficient to account for the diversity of the conformations sampled by hybrid methods.

#### Comparison of Global Modes/Principal Directions of Motion

**FIGURE 5:**
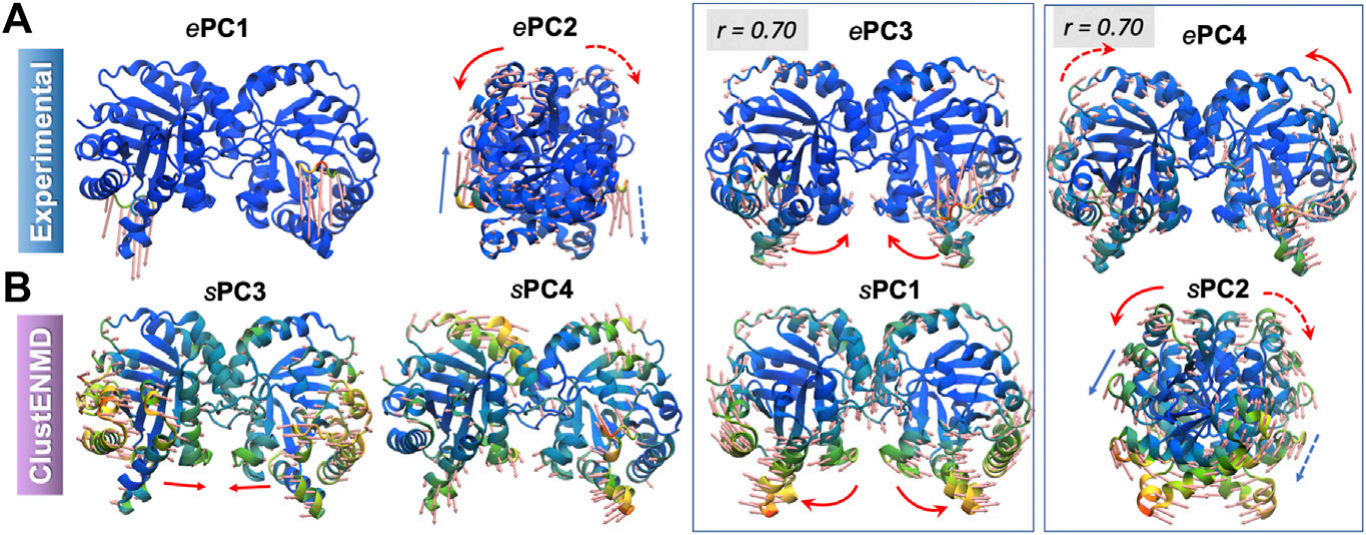
First four principal modes for TIM ensembles. The **(A,B)** refer to experimental and ClustENMD ensembles, respectively. Those PCs that exhibit high (>0.70) correlations are enclosed in boxes. See the corresponding movies in Supplementary Movie S2.

Supplementary Figure S2 shows the overlaps between the top four PCs for each pair of ensembles. The *s*PCs of the hybrid methods are in close agreement with each other, and also in accord with the first three *s*PCs from MD. As discussed above, the first two *e*PCs do not show significant correlation with the *s*PCs (*bottom two rows*), whereas higher overlaps are evident between *e*PCs 3–4 and *s*PCs 1–2. Not surprisingly, RWSIP values are generally relatively low (0.53–0.58) for this enzyme. Closer examination shows that, *e*PC1 primarily reflects the catalytic loop opening/closure (Figure 5), also evident from the large distance change in the loop shown in Figure 4D. As such, it is a *local* motion, and it is not among the *s*PC1-4 that the hybrid methods yield (top-ranking *s*PCs usually describe cooperative motions that engage the *entire* protein). *e*PC2, on the other hand, refers to loop motions coupled to relatively more collective motions and shows slightly higher, but still weak, correlations with *s*PCs. In contrast, *e*PC3 and *e*PC4 do exhibit notable correlations with *s*PC1 or *s*PC2 from all simulations. These motions correspond to the counter-rotation and bending of the subunits with respect to each other, coupled to the catalytic loop dynamics, as illustrated in Figure 5 and Supplementary Movie S2. These motions have been identified as the global modes that define the enzyme’s putative functional motions (Kurkcuoglu et al., 2006; Cansu and Doruker, 2008; Kurkcuoglu et al., 2015). Notably, *s*PC3 is another highly cooperative motion where the two monomers concertedly bend around an axis perpendicular to that of *s*PC1; and *s*PC4 exhibits a counter-twisting and breathing of the two barrels (Figure 5). Overall, hybrid methods point to a broad range of cooperative rearrangements, which cannot be readily discerned upon PCA of structures resolved for TIM homologues which yields either local loop motions (ePC1-2) or highly constrained (small amplitude) global motions (*e*PC3-4).

### 3-Phosphoglycerate Kinase

*PGK* is another key glycolytic enzyme, catalyzing the phospho-transfer between 1,3-bisphosphoglycerate (bPG) and ADP. It is a monomeric protein composed of two domains of approximately equal size. The bPG binding site is located on the N-domain, while ADP binds to the C-domain (Figure 1D). During its function, the enzyme undergoes a large hinge-bending conformational change bringing the bound substrates into proximity such that the reaction can happen (Palmai et al., 2009; Palmai et al., 2014). The open crystal structure in complex with 3-phosphoglyceric acid (3 PG) and ADP [PDB id: 2xe7 (Zerrad et al., 2011)] is used here as the reference structure for initiating the computations.

#### Conformational Variability From Experiments and Computations

**FIGURE 6:**
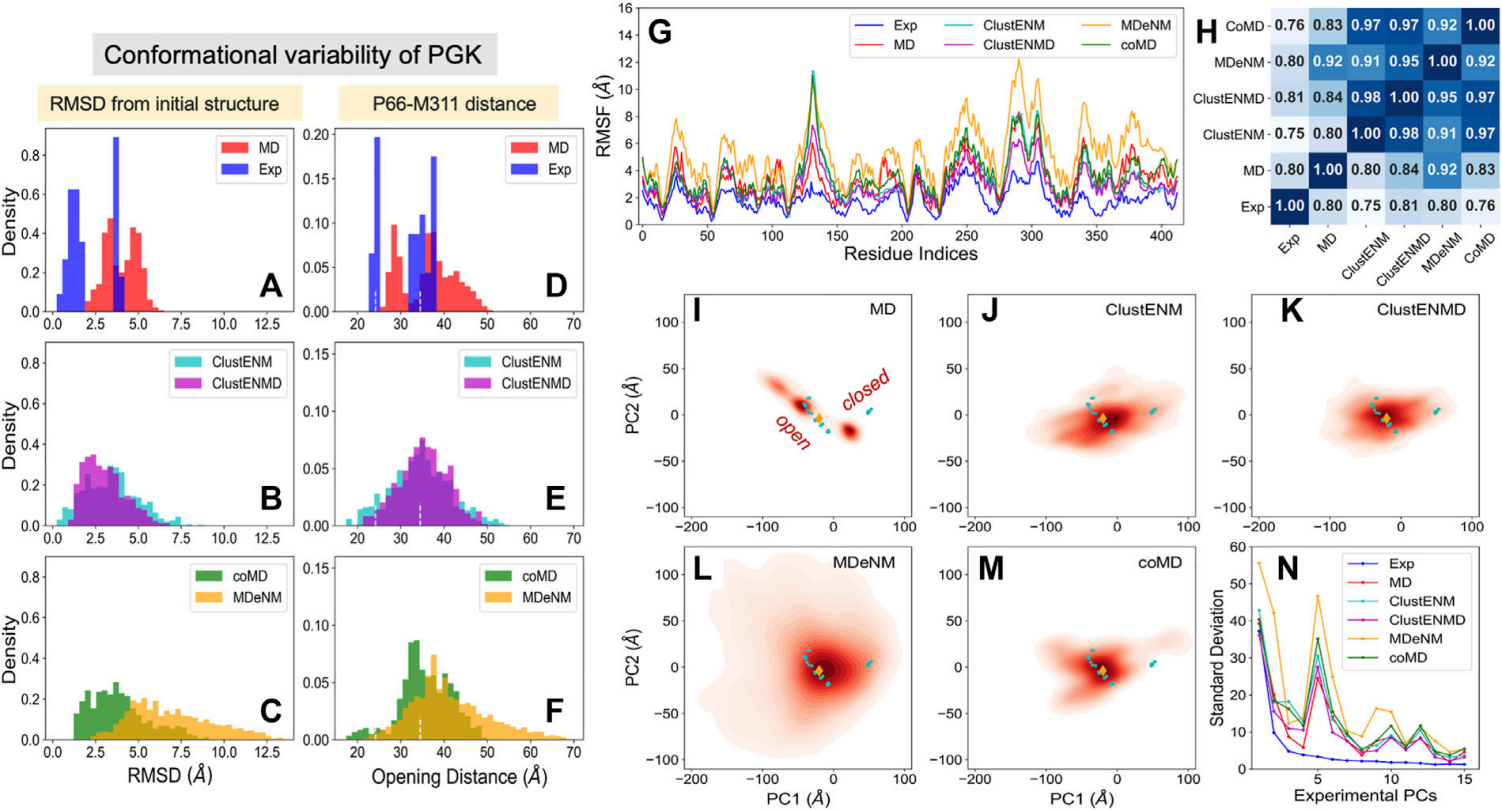
Comparison of computational and experimental ensembles of PGK. The panels are in the same format as Figure 2 **(A–C**) RMSDs with respect to the starting open structure (PDB id: 2xe7). **(D–F)** Distances between the α-carbons of P66 and M311 that monitors the opening-closing motion of PGK. Dashed lines indicate the distances of the initial open, and a catalytically fully closed crystal structure (PDB id: 2wzb). **(G)** RMSFs for each ensemble**, (H)** Pearson correlation coefficients between all pairs of RMSF profiles. **(I–M)** Population distributions of ensembles of conformers generated by simulations, projected onto the subspace spanned by *e*PC1 and *e*PC2, shown for **(I)** MD simulations, **(J)** ClustENM, **(K)** ClustENMD, **(L)** MDeNM, and **(M)** coMD. **(N**) Standard deviations of the conformers projected along the experimental PCs.

We considered 35 experimentally resolved structures for PGK, with sequence identity above 90%. Figures 6A–C shows the RMSDs of the different conformational ensembles, including the ensemble of experimentally resolved structures and conformers from MD simulations (panel **A**), and those generated by hybrid methods (panels **B–C**) with respect to the initial open structure. The conformers were superposed onto the mean experimental structure using the C^α^ coordinates in both domains. There are two separate groups of experimentally resolved structures (Figure 6A), with the lower RMSD group corresponding to the open structures, and that centered around 3.7 Å corresponding to closed structures. All hybrid methods yielded wide unimodal distributions for RMSDs in contrast to the bimodal distributions exhibited by experimental structures and MD conformers. Larger RMSD regions correspond to further opening/relaxation of the protein, beyond that observed in the crystals and/or accessed in MD simulations.

The opening-closing motion of PGK can be monitored through the distance between the α-carbons of P66 and M311, two residues located at the tips of the N- and C-domains in the vicinity of the ligands. Figures 6D–F provides the distributions of the interdomain distance probed by these two residues. For reference, the distance in the initial open structure (34.5 Å) and that assumed in a catalytically active fully closed crystal structure (PDB id: 2wzb (Cliff et al., 2010), 24.2 Å) are indicated by the *white dashed lines*. Figure 6D clearly distinguishes between the closed and open experimental structures. The MD conformers exhibit further opening as well as moderate closing of PGK but do not cover the region of fully closed experimental structures. On the other hand, all hybrid methods successfully detect the fully closed region albeit to different extents. In contrast to the other simulation methods, MD, coMD, and MDeNM included ADP and 3 PG in the binding pocket. This hindered the sampling of conformations beyond the fully closed experimental structures (under 23 Å), while ClustENM and ClustENMD suggested that the interdomain distance could become lower than 20 Å. CoMD, which included the ligands in the TMD and EM stages but not in the C^α^-based ANM for the NMA, still sampled these conformations to a small extent.

#### Residue Fluctuation Profiles

The RMSF in α-carbons with respect to their mean positions are presented in Figure 6G. Like HIV-1 PR and TIM, computations yielded higher fluctuations compared to experiments. In contrast to Figure 4G for TIM, the MD-generated ensemble for PGK exhibited RMSFs falling within the same range as for the hybrid methods. This could be due to the bimodal distribution of the opening distances (Figure 6D) displayed by the three independent MD runs. In contrast, the hybrid methods showed broad unimodal distributions (Figures 6E,F). Yet, the predicted RMSF profiles (Figure 6G) remained comparable to that obtained by MD. We note that MDeNM (*yellow curve*) yielded the largest fluctuations, consistent with the sampling of widely open conformers (see the corresponding long tails in panels **C** and **F**); however, as shown in Figure 6G, the overall profile indicated by experiments and MD simulations were robustly reproduced by all hybrid methods. The pairwise Pearson correlation coefficients presented in Figure 6H show that all hybrid methods exhibited a fairly strong correlation among themselves (varying from 0.91 to 0.98), similar to the results observed in HIV-1 PR and TIM. This time, we also observe a relatively strong correlation between the hybrid methods and experiments (0.75–0.81) and MD runs (0.80–0.92). MDeNM exhibits a remarkably high correlation (0.92) with MD revealing that the relative flexibilities of the residues are accurately accounted for, even though the absolute sizes of the motions (uniformly and significantly) differ.

#### Conformational Landscape

Figures 6I–M display the projections of the computationally generated conformers onto the reduced space spanned by *e*PC1 and *e*PC2. Two distinct sets of experimentally resolved structures (*cyan dots*) are discerned: one corresponding to open structures (including the reference structure denoted by the *orange diamond*), the other to closed structures. MD simulations starting from the open structure could not sample the region of closed conformations, and only partially covered the space sampled by the open experimental structures. All hybrid methods, on the other hand, covered the space occupied by both groups of structures. Considerable opening of PGK is visible in ClustENM-generated conformers and an even greater opening is predicted by MDeNM.

#### Comparison of Global Modes/Principal Directions of Motion

Figure 6N describes the standard deviation (or square root of variance) of the conformers along the first 15 *e*PCs. All computationally generated ensembles show a wide dispersion along *e*PC1 and *e*PC5, with *e*PC5 showing a clear peak. Supplementary Figure S3 provides the overlap matrices (first five modes) among each pair of ensembles. The *bottom row* clearly shows that the *s*PC2 predicted by all four hybrid methods strongly correlate with *e*PC1, with correlation cosines ranging from 0.76 (coMD) to 0.89 (MDeNM), hence the broad dispersion of the computationally predicted conformers along *e*PC1 (driven by their *s*PC2). The high variance of predicted conformers along *e*PC5, on the other hand, apparently originates from the overlap with the first mode, *s*PC1, predicted by all hybrid methods. The overlaps are moderate in this case, varying from 0.52 for MDeNM to 0.61 for ClustENM. Notably, the *s*PCs predicted by MD show the weakest correlations with experiments among all computational methods, but still exhibit modest overlaps with *e*PC1 and *e*PC5. Likewise, MD shows the lowest RWSIP in Table 3 while ClustENM and ClustENMD show the highest.

**FIGURE 7:**
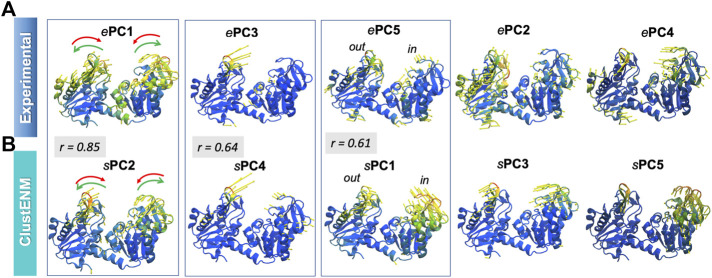
First five principal modes for PGK ensembles. The **(A,B)** are based on PCA of experimental and ClustENM ensembles, respectively. Those pairs of PCs that exhibit relatively high (>0.60) correlations are enclosed in boxes. See the corresponding Supplementary Movie S3.

Figure 7 illustrates, using color-coded ribbon diagrams and arrows, the first five ePCs (*upper panel*) and *s*PCs from ClustENM (*lower panel*). Both experiments and ClustENM describe the opening-closing (hinge-bending) motion as well as some breathing motions of the two domains. *e*PC1, counterpart of *s*PC2 as discussed above, represents the hinge-bending motion mediated by the interdomain helix. *e*PC3 corresponds to large conformational changes at the loop (residues 130–140) located at the tip of the N-domain, also expressed in large RMSF values in Figure 6G. Notably ClustENM *s*PC4 approximates the same movement (with a correlation cosine 0.64), also shown in Figure 7. ClustENM *s*PC1 and its approximate counterpart *e*PC5 induce out-of-plane motions, inward and outward, in the two respective domains, even though the size of motions along *e*PC5 is smaller. Supplementary Movie S3 illustrates the ClustENM *s*PC1, *s*PC2 and *s*PC4, as the three functional mechanisms of motions accessible to PGK.

#### HIV-1 Reverse Transcriptase

Like HIV-1 protease, RT has been, and continues to be, an important target for HIV-1 drug discovery (Esposito et al., 2012; Gu et al., 2020). RT is a heterodimer composed of subunits p66 and p51 (Figure 1C). The p66 subunit contains the DNA polymerase and RNase H domains, thus performing dual enzymatic activity, while the p51 subunit serves as a scaffold. The DNA polymerase domain itself consists of four subdomains: fingers, thumb, palm, and connection. The former two are distinguished by their high mobility required to bind the nucleotide oligomer; the palm serves as a hinge center, and the connection forms the base connecting to the p51 subunit. Nucleoside/nucleotide RT inhibitors (NRTIs) were the first class of antiretroviral drugs approved for therapeutic use, followed by non-nucleoside/nucleotide RT inhibitors (NNRTIs). Most NNRTIs (Namasivayam et al., 2019) bind a pocket at the palm interface with the thumb or fingers, impairing the hinge movements of these two subdomains essential to polymerase activity. Moreover, inhibitors have been designed that control the global movements of the RNAse H (Ilina et al., 2012), and even those having dual actions on both enzymatic activity exist (Corona et al., 2016).

##### Conformational Variability From Experiments and Computations

**FIGURE 8:**
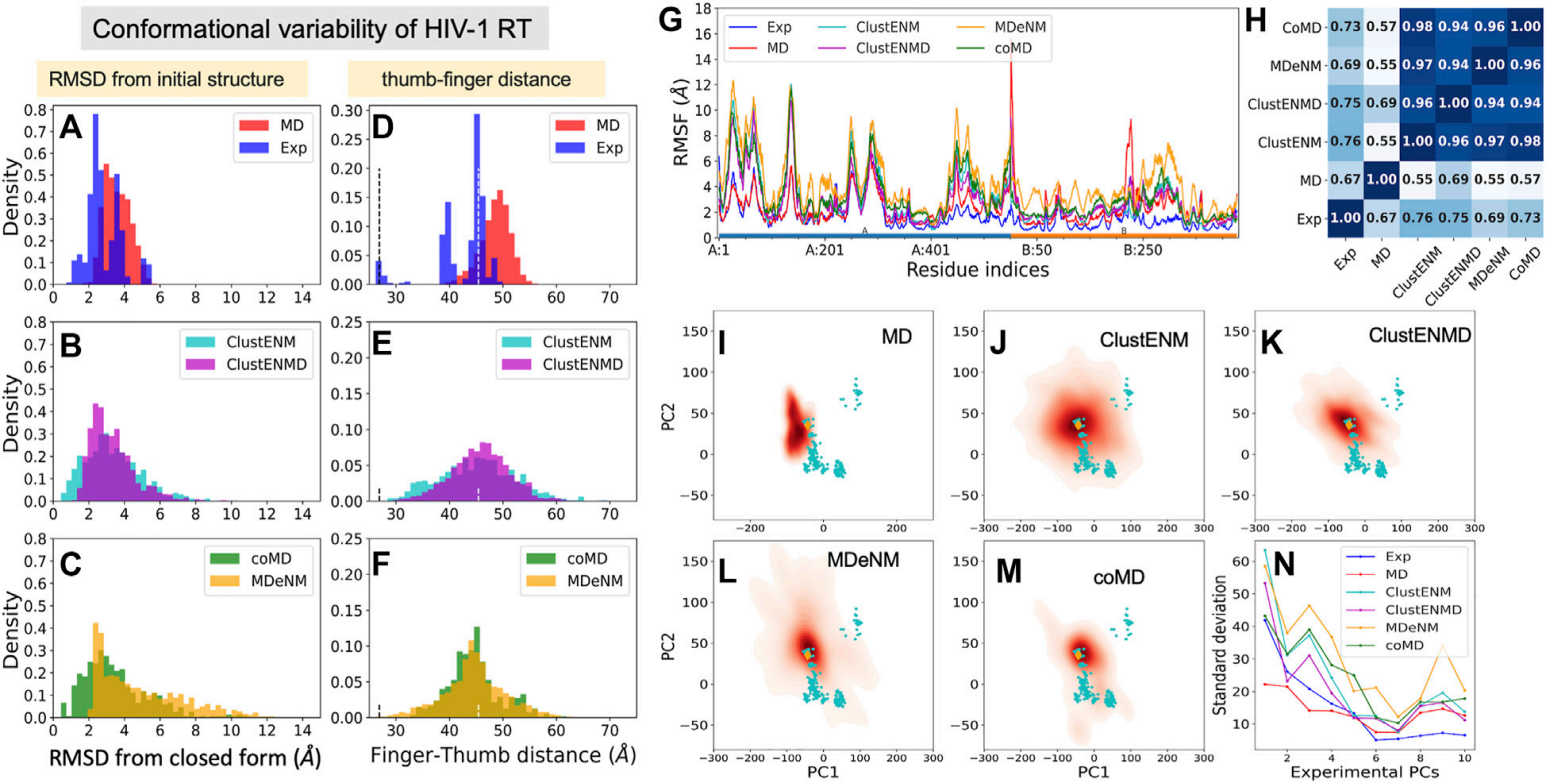
Comparison of computational and experimental ensembles of HIV-1 RT. **(A–C)** RMSDs with respect to the starting closed ligand-free structure (PDB id: 3kli). **(D–F)** Distances between the centers of mass of the fingers (residues 1–85 to 112–155) and thumb (residues 242–312) subdomains on the p66 subunit (chain A) that describe open-to-closed fluctuations of the two subdomains. *Dashed lines* indicate the distances in the open and closed reference structures (PDB id: 2b6a and 3kli, respectively). **(G)** RMSFs for each ensemble. The *bars* underneath indicate the p66 subunit (chain A; *blue*) and the p51 subunit (chain B; *orange*). **(H)** Pearson correlation coefficients between pairs of RMSF profiles. **(I–M)** Population distributions for ensembles generated by **(I)** MD, **(J)** ClustENM, **(K)** ClustENMD, **(L)** MDeNM, and **(M)** coMD. **(N)** Standard deviations of the conformers projected along the experimental PCs.

We used as reference a complex with a NNRTI (THR-50) [PDB id: 2b6a (Morningstar et al., 2007)], Figure 1C, which represents an *open* form of the fingers-thumbs subdomain of RT. Figures 8A–C displays the RMSDs with respect to the closed reference structure [PDB id: 3kli (Tu et al., 2010)] observed in experiments and computations. The resolved structures (365 included here) show a conformational variability (1 ≤ RMSD ≤ 6 Å) wider than those of the other three proteins studied, and the hybrid methods show even broader distributions. MD simulations show the narrowest distributions (2 ≤ RMSD ≤ 6 Å), unable to sample the closed forms. MDeNM shows the highest RMSDs (up to 12 Å) but cannot sample the closed forms with RMSD < 2 Å while ClustENM and coMD satisfactorily sample both closed and open forms (Figures 8A–C). The ability of ENM-based hybrid methods to sample the broad range of subdomain and domain rearrangements of RT originates from the ability of ENMs to describe the RT global dynamics (Bahar et al., 1999; Sluis-Cremer et al., 2004).

Toward understanding the origin of these large RMSDs, we examined the distance between the mass centers of the thumb and fingers subdomains (Figures 8D–F), which is a determinant of conformational variability. The *vertical dashed lines* indicate the distances corresponding to the closed and open states (*d* = 26.9 and 45.4 Å, respectively). As noted above, MD conformers only sample open conformers (within 40 ≤ *d* ≤55 Å), whereas the hybrid methods exhibit broader distributions encompassing a wide distribution of thumb-finder distances.

##### Residue Fluctuation Profiles

Residue fluctuation profiles are presented in Figure 8G, along with their Pearson correlation coefficients in panel H. Despite their large RMSFs, RMSF profiles of all hybrid methods as well as MD simulations and experiments show close similarities. The correlations of the hybrid methods with experiments vary from 0.69 (MDeNM) to 0.76 (ClustENM), while those with MD are lower (0.55–0.69). This, and the lower correlation between MD and experiments (0.67), indicates that the large movements undergone by the experimental structures adhere to the intrinsic dynamics of RT constrained by its overall fold topology as predicted by hybrid methods, while MD simulations of 200 ns fall short of an adequate sampling of conformational space for this large (1,000 residues) protein.

##### Conformational Landscape

The results are shown in Figures 8I–N, in the same format as before. Hybrid methods show sampling power superior to that of MD: ClustENM (panel **J**), coMD (panel **M**) and MDeNM (panel **L**) cover a space large enough to include a significant share of experimental structures (*cyan dots*) in contrast to MD. Notably, the space sampled by ClustENM encompasses almost all experimental structures projected onto the subspace spanned by *e*PC1 and 2.

##### Comparison of Global Modes/Principal Directions of Motion

Figure 8N describes the standard deviation (or square root of variance) of the conformers along the first 10 *e*PCs. The computationally generated ensembles excluding MD show a wide dispersion along *e*PC1 to *e*PC4, supporting the wide diversity of the generated structures. ClustENM and MDeNM standout as the two hybrid methods that yield the largest dispersion of conformers along *e*PC1-2. ClustENM also had the highest RWSIP value (0.72; Table 3), while MD yields the lowest (0.43).

**FIGURE 9:**
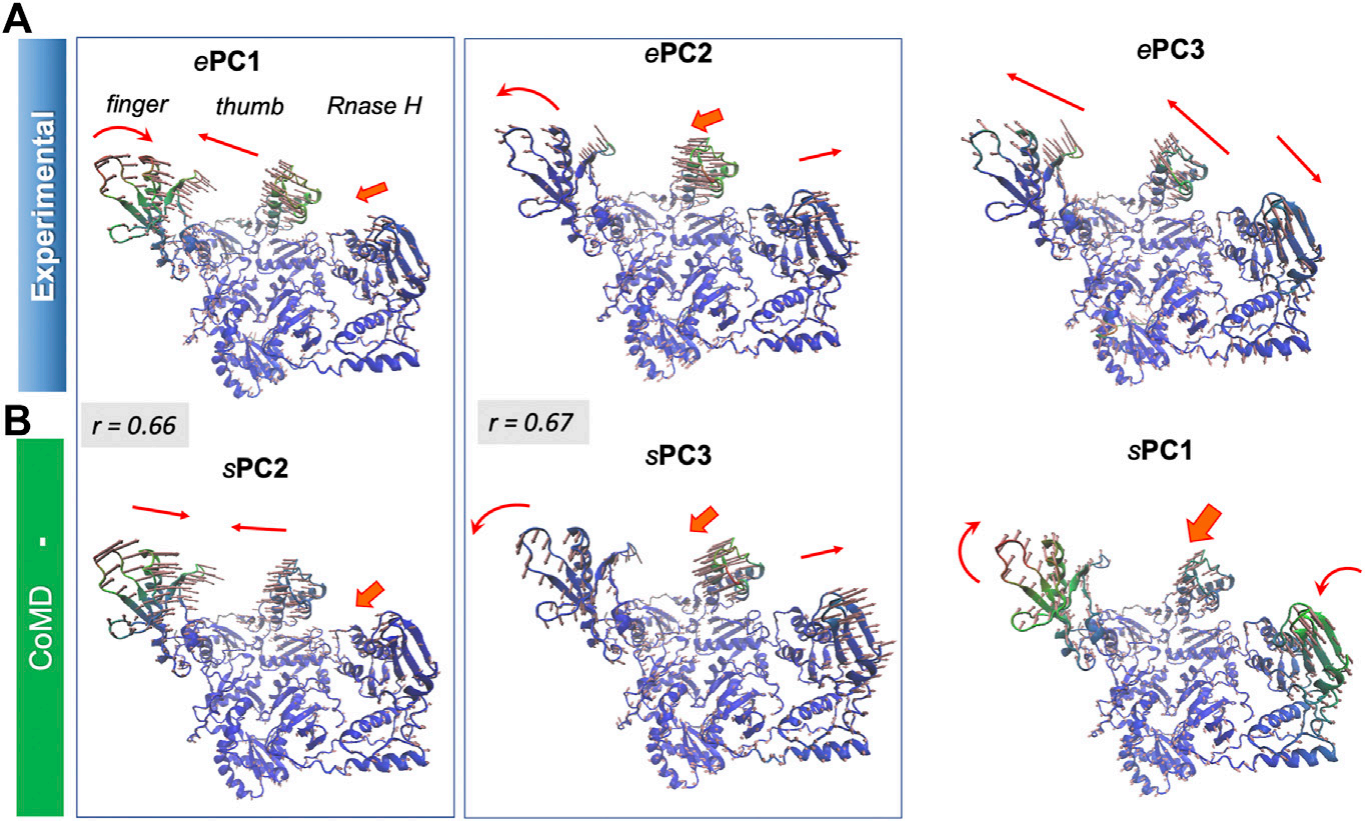
First three principal modes for HIV-1 RT ensembles. The **(A,B)** are based on PCA of experimental and coMD ensembles, respectively. Pairs that exhibit relatively high (>0.65) correlations are enclosed in boxes. See the corresponding Supplementary Movie S4.

Supplementary Figure S4 provides the overlap matrix between the top four PCs for all pairs of conformational ensembles. The *bottom row* shows that *e*PC1 correlates with the *s*PC2 of all four hybrid methods, with correlation cosines varying from 0.59 (MDeNM) to 0.66 (coMD and ClustENM), in addition to *s*PC1 of ClustENMD (0.58) and ClustENM (0.56) and *s*PC3 from MDeNM (0.57). Figure 9 and Supplementary Movie S4 show that this PC describes the opening-closing of the thumb and finger subdomains with respect to each other, accompanied by concerted reorientation of RNase H domain. Likewise, *e*PC2 shows a good correlation with *s*PC3 (e.g. 0.67 for coMD). In this case, the finger and thumb subdomains of the DNA polymerase domain undergo anticorrelated movements with respect to RNAse H (Figure 9). Notably, the computationally predicted first mode of motion (*s*PC1), which is in remarkably strong agreement between all four hybrid methods (correlation cosines >0.90), is not accounted for by *e*PC1-4. This essential motion (relative movements of the fingers and RNase H accompanied by out-of-plane movements of the thumb), also supported by MD, is also illustrated in Supplementary Movie S4.

## Conclusion

Recent years have seen an increase in the number and complexity of hybrid methods developed for investigating the conformational space accessible to proteins (Krieger et al., 2020), and especially to complexes or multimeric proteins. This has been accompanying the advances in experimental technologies that allow for the elucidation of multiple conformers, and the increasing need to map the accessible conformational space toward elucidating the mechanisms of function. While such hybrid methods appear to provide tools for exploring large-scale conformational motions at atomic resolution, it is of interest to assess their limitations as well as their advantages in a comparative analysis. Here we focused on four such methods and used as benchmarks sets of experimentally resolved structures and MD-sampled conformers for four well-studied proteins. Our analysis simultaneously revealed that these two latter sets suffer from limitations themselves, as discussed below. Overall, six ensembles of conformers were compared in each case, including those observed experimentally, simulated by MD, and predicted by hybrid methods. The analysis used four criteria/metrics, and the performance of the methods vis-à-vis each metric is discussed below.

The overall breadth of conformational space predicted by hybrid methods is significantly larger than that observed in X-ray structures or sampled by MD simulations. In all cases, the RMSDs of the conformers generated by the hybrid methods exhibited a much broader distribution than those experimentally observed, as illustrated in panels **A-C** of Figure 2, Figure 4, Figure 6, and Figure 8. This is most striking in the TIM analysis. Despite the inclusion of TIM sequence homologs with >50% sequence identity, the maximum RMSD between these 57 structures remained 1.1 Å (Figure 4A). Thus, the crystal structures resolved for this dimeric enzyme exhibit minimal global conformational variability, which is presumably partly due to constraints in the crystal environment and partly to the particular highly stable α/β-barrel fold. Local functional changes evidenced by large fluctuations in inter-residue distances (G174-Y211) are observed in this case with minimal domain/monomer movements. The RMSD of conformers predicted by ClustENMD and coMD remained generally lower than 4 Å with respect to the initial structure, which is plausible for a dimeric enzyme of ∼500 residues. MDeNM and ClustENM, on the other hand, led to up to 8 Å RMSDs, and it remains to be seen if such large conformational changes are accessible to TIM family members. In contrast, the X-ray structures for RT showed a much broader variation (up to 6 Å RMSD), and all hybrid methods satisfactorily reproduced the breadth of conformational variation (Figures 8A–C).

Hybrid methods predict conformers that comply with functional changes in conformations. For each studied system, we selected specific distances that probe functional movements (e.g. flap opening/closure in PR, thumb-finger distance in RT, catalytic loop motion in TIM, interdomain distance in PGK) and investigated whether hybrid methods could produce conformers consistent with experimentally detected changes (Figure 2, Figure 4, Figure 6 and Figure 8; panels D–F**)**. The challenge in most simulations is to capture closed conformations, or the closest distances, which are not entropically favored. Table 3 lists these closest distances of approach for residue pairs selected to reflect functional movements (*first column*), observed in computations and experiments. Except for RT, the hybrid methods yielded conformers that satisfied the closest distances of approach, even exceeding in some cases the closest distances observed in experiments. In the case of RT, the closest thumb-fingers distance observed in experiments is captured by MDeNM, and approached by ClustENM and ClustENMD but not by coMD and MD. We note that MD also failed to sample the closest interdomain distances in PGK; and MDeNM could sample only the extended forms of TIM catalytic loop.

As another metric we examined whether the closed form could be attained when initiating the simulations from the open form. RMSDs between the reference open and closed forms of PR, PGK and RT were 2.7, 3.6 and 5.2A, respectively. Hybrid methods demonstrated a substantially higher ability to attain the closed state compared to MD simulations (Table 3).

Residue fluctuations (RMSFs) exhibit robust profiles, despite significant (uniformly distributed) changes in the overall sizes of motions. A striking observation repeatedly observed in all four proteins and quantified by Pearson correlations (of >0.86) was the robustness of RMSF profiles predicted by all four hybrid methods, despite their differences in the absolute RMSFs (Figure 2, Figure 4, Figure 6 and Figure 8, panels G-H). Even more interesting was their strong correlation with the RMSF profiles extracted from aligned experimental structures, despite the significant suppression of fluctuations in crystal structures. For HIV-1 PR, the correlations between experimental RMSFs and those predicted by hybrid methods fell in the range 0.92 ± 0.02; for PGK and RT, they vary as 0.78 ± 0.03 and 0.73 ± 0.03. In contrast, TIM exhibited significantly lower correlations (0.56 ± 0.04). As pointed out above, the ensembles of structures resolved for TIM are very narrowly distributed, and so are the residue RMSFs. The RMSFs extracted from these highly similar crystal structures may not reflect the full conformational spectrum.

The correlations between RMSF profiles predicted by hybrid methods and MD simulations, varied in the ranges 0.88 ± 0.09, 0.74 ± 0.02, 0.86 ± 0.06 for PR, TIM and PGK, respectively, while that of RT was much lower (0.59 ± 0.09). Given that hybrid results gave a significantly higher correlation with experiments, this low correlation indicates the sampling inaccuracy of MD (of 100 s of nanoseconds) for this protein of 1,000 residues. Finally, the comparison of the Pearson correlations between experimental and simulated RMSFs showed that ClustENMD and MDeNM simulations achieved slightly higher performances (0.77 and 0.75 respectively, averaged over the four case studies), followed by coMD, MD and ClustENM which showed comparable performance (∼0.74).

Closer look at principal changes in conformation points to the conservation of dominant modes of motion, supported by both experiments and computations. Dissection of the spectrum of collective motions upon PCA of the generated conformers in each ensemble revealed close similarities, as shown in the overlap matrices presented in Supplementary Figures S1–S4 for top-ranking 4 PCs (or 5 for PGK). Given that PCs usually define cooperative mechanisms relevant to function, it is important to assess to what extent the *s*PCs derived from hybrid methods agree with experimental PCs (*e*PCs). Using RWSIP (Carnevale et al., 2007) as a metric (Table 3), we found that ClustENMD performed the best among the examined five computational methods, followed by ClustENM and MDeNM. These two analyses also allowed us to identify commonalities and divergences between methods to reveal the most salient features for each system, revealing the benefit of using multiple methods together.

We also observed significant variations of generated conformers along experimental PCs other than *e*PC1 and *e*PC2 (panel **N** in Figure 2, Figure 4, Figure 6 and Figure 8). For example, TIM shows higher variations along the third and fourth *e*PCs compared to those along the first two *e*PCs in all simulations including MD. Likewise, an essential mechanism of motion of RT, robustly predicted as *s*PC1 by all computational methods and known to be essential to function (Jernigan et al., 2000; Sluis-Cremer et al., 2004) eludes the top-ranking *e*PCs. These observations show some global modes/deformations are suppressed in the X-ray structures presumably due to tight packing or symmetry requirements imposed by crystallization.

Other considerations: Optimization of parameter sets and computing efficiency. The parameters used in ClustENM, ClustENMD, and coMD were the same across all the systems, where those of MDeNM were adjusted for different proteins (Table 2). These parameters for the former three methods seem to perform satisfactorily for the studied proteins, except for RT in terms of reaching the closed structure (e.g., PDB id: 3kli) starting from an open form. If the extent of conformational flexibility (e.g. RMSD between endpoints) were to be known in advance, the parameters could be adjusted for more precise sampling of conformers. Obviously, generation of additional cycles enables the sampling of a broader conformational space, which may be more appropriate for larger proteins. A systematic study of the size of experimentally observed conformational space as a function of the size and packing density of protein, or different structural classes may provide guidance for selecting parameters based on the system properties. Given that the hybrid methods tested here are relatively recent, there is plenty of room for subsequent studies aiming to define optimal parameters for different systems.

To provide initial insights into the influence of these sampling parameters, we analyzed the progression of RWSIP values as a function of the number of generations/cycles/excitations depending upon the hybrid method for PGK as an illustrative case. Supplementary Figure S5 shows the progression of RWSIP values as a function of the number of generations for ClustENM and ClustENMD, where the conformers of the current generation are added to the ensemble of previous ones in each successive generation. The RWSIP values of the three independent runs, the average values, as well as the values of the combined ensemble comprising all three runs, are observed to start converging after the second generation and saturate in both cases. This indicates that the intrinsic dynamics encoded by the experimental structures is achieved in early generations. However, the conformers obtained in the later generations allow for approaching the closed conformer of PGK.

Supplementary Figure S6 shows the equivalent progression of RWSIP values for MDeNM. The number of excitations does not influence the RWSIP values in this case, as the same directions of motion are excited each time for any given replica, but the number of excitations is again important for the degree of conformational space sampled including the approach towards closed conformers. The number of replicas is a key parameter that determines the directional coverage and the RWSIP value rises with the number of replicas. Some transition points are observed at about 2, 5 and 10 replicas along with a slow convergence after 30 to 40 replicas.

Supplementary Figure S7 shows the equivalent progression of RWSIP values for coMD. In this case, the direction changes every cycle, and the number of cycles thus makes a much bigger difference. Interestingly, we observe two convergence regimes. Firstly, about 15 cycles is required to converge upon an optimal RWSIP value. However, after about 25–30 cycles, this value decreases as additional directions are explored and the RWSP converges on a new, lower value. The RWSIP is therefore a useful criterion for assessing how many cycles are beneficial for coMD, just like the number of replicas for MDeNM. Looking at how the RWSIP changes as a function of the number of runs, it is clear that there can be substantial variation between runs with some having much higher RWSIP values than others. It is therefore necessary to include a large enough number of runs (e.g., 5 or more) to obtain a sufficient coverage of motion directions.

Finally, an important advantage of hybrid methods is their computational efficiency, and this is without compromising their accuracy as the current comparison with experimental and MD data demonstrates. In particular, the efficiency of ClustENM and ClustENMD are reflected by run times on the order of minutes (Supplementary Table S1), while coMD is of the order of hours. The computational efficiency of ClustENM and ClustENMD stems from the usage of implicit solvent during the EM or MD steps, in addition to the adoption of ENMs for predicting the NMs. Given that ENM-based methods provide accuracy levels comparable to those based on full atomic models (MDeNM and MD) with significant savings in computing time, further development of MDeNM methodology using ENM-based NMA, as opposed to full atomic NMA, is currently in progress.

## Data Availability

The raw data supporting the conclusion of this article will be made available by the authors, without undue reservation.

## References

[B1] Atilgan A. R. Durell S. R. Jernigan R. L. Demirel M. C. Keskin O. Bahar I. (2001). Anisotropy of Fluctuation Dynamics of Proteins with an Elastic Network Model. Biophysical J. 80, 505–515. 10.1016/s0006-3495(01)76033-x PMC1301252 11159421

[B2] Badaya A. Sasidhar Y. U. (2020). Inhibition of the Activity of HIV-1 Protease through Antibody Binding and Mutations Probed by Molecular Dynamics Simulations. Sci. Rep. 10, 5501. 10.1038/s41598-020-62423-y 32218488 PMC7098958

[B3] Bahar I. Erman B. Jernigan R. L. Atilgan A. R. Covell D. G. (1999). Collective Motions in HIV-1 Reverse Transcriptase: Examination of Flexibility and Enzyme Function. J. Mol. Biol. 285, 1023–1037. 10.1006/jmbi.1998.2371 9887265

[B4] Bahar I. Lezon T. R. Yang L.-W. Eyal E. (2010). Global Dynamics of Proteins: Bridging between Structure and Function. Annu. Rev. Biophys. 39, 23–42. 10.1146/annurev.biophys.093008.131258 20192781 PMC2938190

[B5] Bakan A. Bahar I. (2009). The Intrinsic Dynamics of Enzymes Plays a Dominant Role in Determining the Structural Changes Induced upon Inhibitor Binding. Proc. Natl. Acad. Sci. 106, 14349–14354. 10.1073/pnas.0904214106 19706521 PMC2728110

[B6] Bakan A. Dutta A. Mao W. Liu Y. Chennubhotla C. Lezon T. R. (2014). Evol and ProDy for Bridging Protein Sequence Evolution and Structural Dynamics. Bioinformatics 30, 2681–2683. 10.1093/bioinformatics/btu336 24849577 PMC4155247

[B7] Bakan A. Meireles L. M. Bahar I. (2011). ProDy: Protein Dynamics Inferred from Theory and Experiments. Bioinformatics 27, 1575–1577. 10.1093/bioinformatics/btr168 21471012 PMC3102222

[B8] Batista P. R. Pandey G. Pascutti P. G. Bisch P. M. Perahia D. Robert C. H. (2011). Free Energy Profiles along Consensus Normal Modes Provide Insight into HIV-1 Protease Flap Opening. J. Chem. Theor. Comput. 7, 2348–2352. 10.1021/ct200237u 26606609

[B9] Brändén G. Neutze R. (2021). Advances and Challenges in Time-Resolved Macromolecular Crystallography. Science 373. 10.1126/science.aba0954 34446579

[B10] Brooks B. R. Brooks C. L. Mackerell A. D. Nilsson L. Petrella R. J. Roux B. (2009). CHARMM: the Biomolecular Simulation Program. J. Comput. Chem. 30, 1545–1614. 10.1002/jcc.21287 19444816 PMC2810661

[B11] Can M. T. Kurkcuoglu Z. Ezeroglu G. Uyar A. Kurkcuoglu O. Doruker P. (2017). Conformational Dynamics of Bacterial Trigger Factor in Apo and Ribosome-Bound States. PLoS One 12, e0176262. 10.1371/journal.pone.0176262 28437479 PMC5402958

[B12] Cansu S. Doruker P. (2008). Dimerization Affects Collective Dynamics of Triosephosphate Isomerase. Biochemistry 47, 1358–1368. 10.1021/bi701916b 18189421

[B13] Carnevale V. Pontiggia F. Micheletti C. (2007). Structural and Dynamical Alignment of Enzymes with Partial Structural Similarity. J. Phys. Condens. Matter 19, 285206. 10.1088/0953-8984/19/28/285206

[B83] Cliff M. J. Bowler M. W. Varga A. Marston J. P. Szabo J. Hounslow A. M. (2010). Transition State Analogue Structures of Human Phosphoglycerate Kinase Establish the Importance of Charge Balance in Catalysis. J. Am. Chem. Soc. 132 (18), 6507–6516. 10.1021/ja100974t 20397725

[B14] Corona A. Meleddu R. Esposito F. Distinto S. Bianco G. Masaoka T. (2016). Ribonuclease H/DNA Polymerase HIV-1 Reverse Transcriptase Dual Inhibitor: Mechanistic Studies on the Allosteric Mode of Action of Isatin-Based Compound RMNC6. PLoS One 11, e0147225. 10.1371/journal.pone.0147225 26800261 PMC4723341

[B15] Costa M. G. S. Batista P. R. Bisch P. M. Perahia D. (2015). Exploring Free Energy Landscapes of Large Conformational Changes: Molecular Dynamics with Excited normal Modes. J. Chem. Theor. Comput. 11, 2755–2767. 10.1021/acs.jctc.5b00003 26575568

[B16] Costa M. G. S. Fagnen C. Vénien-Bryan C. Perahia D. (2020). A New Strategy for Atomic Flexible Fitting in Cryo-EM Maps by Molecular Dynamics with Excited Normal Modes (MDeNM-EMfit). J. Chem. Inf. Model. 60, 2419–2423. 10.1021/acs.jcim.9b01148 31944765

[B17] Dudas B. Merzel F. Jang H. Nussinov R. Perahia D. Balog E. (2020). Nucleotide-Specific Autoinhibition of Full-Length K-Ras4B Identified by Extensive Conformational Sampling. Front. Mol. Biosci. 7, 145. 10.3389/fmolb.2020.00145 32754617 PMC7366858

[B18] Dudas B. Perahia D. Balog E. (2021a). Revealing the Activation Mechanism of Autoinhibited RalF by Integrated Simulation and Experimental Approaches. Sci. Rep. 11, 10059. 10.1038/s41598-021-89169-5 33980916 PMC8115643

[B19] Dudas B. Toth D. Perahia D. Nicot A. B. Balog E. Miteva M. A. (2021b). Insights into the Substrate Binding Mechanism of SULT1A1 through Molecular Dynamics with Excited normal Modes Simulations. Sci. Rep. 11, 13129. 10.1038/s41598-021-92480-w 34162941 PMC8222352

[B20] Eastman P. Swails J. Chodera J. D. McGibbon R. T. Zhao Y. Beauchamp K. A. (2017). OpenMM 7: Rapid Development of High Performance Algorithms for Molecular Dynamics. Plos Comput. Biol. 13, e1005659. 10.1371/journal.pcbi.1005659 28746339 PMC5549999

[B21] Esposito F. Corona A. Tramontano E. (2012). HIV-1 Reverse Transcriptase Still Remains a New Drug Target: Structure, Function, Classical Inhibitors, and New Inhibitors with Innovative Mechanisms of Actions. Mol. Biol. Int. 2012, 586401. 10.1155/2012/586401 22778958 PMC3388302

[B22] Eyal E. Lum G. Bahar I. (2015). The Anisotropic Network Model Web Server at 2015 (ANM 2.0). Bioinformatics 31, 1487–1489. 10.1093/bioinformatics/btu847 25568280 PMC4410662

[B23] Fagnen C. Bannwarth L. Oubella I. Forest E. De Zorzi R. de Araujo A. (2020). New Structural Insights into Kir Channel Gating from Molecular Simulations, HDX-MS and Functional Studies. Sci. Rep. 10, 8392. 10.1038/s41598-020-65246-z 32439887 PMC7242327

[B24] Fagnen C. Bannwarth L. Zuniga D. Oubella I. Zorzi R. D. Forest E. (2021). Unexpected Gating Behaviour of an Engineered Potassium Channel Kir. Front. Mol Biosci 8, 538. 10.3389/fmolb.2021.691901 PMC8222812 34179097

[B25] Floquet N. Costa M. G. S. Batista P. R. Renault P. Bisch P. M. Raussin F. (2015). Conformational Equilibrium of CDK/Cyclin Complexes by Molecular Dynamics with Excited Normal Modes. Biophysical J. 109, 1179–1189. 10.1016/j.bpj.2015.07.003 PMC4576171 26255588

[B26] Fuglebakk E. Reuter N. Hinsen K. (2013). Evaluation of Protein Elastic Network Models Based on an Analysis of Collective Motions. J. Chem. Theor. Comput. 9, 5618–5628. 10.1021/ct400399x 26592296

[B27] Gu S.-X. Zhu Y.-Y. Wang C. Wang H.-F. Liu G.-Y. Cao S. (2020). Recent Discoveries in HIV-1 Reverse Transcriptase Inhibitors. Curr. Opin. Pharmacol. 54, 166–172. 10.1016/j.coph.2020.09.017 33176248

[B28] Gur M. Madura J. D. Bahar I. (2013). Global Transitions of Proteins Explored by a Multiscale Hybrid Methodology: Application to Adenylate Kinase. Biophysical J. 105, 1643–1652. 10.1016/j.bpj.2013.07.058 PMC3791301 24094405

[B29] Gur M. Zomot E. Cheng M. H. Bahar I. (2015). Energy Landscape of LeuT from Molecular Simulations. J. Chem. Phys. 143, 243134. 10.1063/1.4936133 26723619 PMC4662675

[B30] Haliloglu T. Bahar I. (2015). Adaptability of Protein Structures to Enable Functional Interactions and Evolutionary Implications. Curr. Opin. Struct. Biol. 35, 17–23. 10.1016/j.sbi.2015.07.007 26254902 PMC4688206

[B31] Henzler-Wildman K. A. Thai V. Lei M. Ott M. Wolf-Watz M. Fenn T. (2007). Intrinsic Motions along an Enzymatic Reaction Trajectory. Nature 450, 838–844. 10.1038/nature06410 18026086

[B32] Hinsen K. (1998). Analysis of Domain Motions by Approximate normal Mode Calculations. Proteins 33, 417–429. 10.1002/(sici)1097-0134(19981115)33:3<417:aid-prot10>3.0.co;2-8 9829700

[B33] Huang J. MacKerell A. D. Jr. (2013). CHARMM36 All-Atom Additive Protein Force Field: Validation Based on Comparison to NMR Data. J. Comput. Chem. 34, 2135–2145. 10.1002/jcc.23354 23832629 PMC3800559

[B34] Huang J. Rauscher S. Nawrocki G. Ran T. Feig M. de Groot B. L. (2017). CHARMM36m: an Improved Force Field for Folded and Intrinsically Disordered Proteins. Nat. Methods 14, 71–73. 10.1038/nmeth.4067 27819658 PMC5199616

[B35] Humphrey W. Dalke A. Schulten K. (1996). VMD: Visual Molecular Dynamics. J. Mol. Graph 14 (33-38), 33–38. 10.1016/0263-7855(96)00018-5 8744570

[B36] Hunter J. D. (2007). Matplotlib: A 2D Graphics Environment. Comput. Sci. Eng. 9, 90–95. 10.1109/mcse.2007.55

[B37] Ilina T. LaBarge K. Sarafianos S. G. Ishima R. Parniak M. A. (2012). Inhibitors of HIV-1 Reverse Transcriptase-Associated Ribonuclease H Activity. Biology 1, 521–541. 10.3390/biology1030521 23599900 PMC3627382

[B38] Jernigan R. L. Bahar I. Covell D. G. Atilgan A. R. Erman B. Flatow D. T. (2000). Relating the Structure ofHIV-1 Reverse Transcriptaseto its Processing Step. J. Biomol. Struct. Dyn. 17, 49–55. 10.1080/07391102.2000.10506603 22607406

[B39] Jiang J. Shrivastava I. H. Watts S. D. Bahar I. Amara S. G. (2011). Large Collective Motions Regulate the Functional Properties of Glutamate Transporter Trimers. Proc. Natl. Acad. Sci. 108, 15141–15146. 10.1073/pnas.1112216108 21876140 PMC3174670

[B40] Jo S. Kim T. Iyer V. G. Im W. (2008). CHARMM-GUI: a Web-Based Graphical User Interface for CHARMM. J. Comput. Chem. 29, 1859–1865. 10.1002/jcc.20945 18351591

[B41] Kaynak B. T. Doruker P. (2019). Protein-Ligand Complexes as Constrained Dynamical Systems. J. Chem. Inf. Model. 59, 2352–2358. 10.1021/acs.jcim.8b00946 30912658

[B42] Kaynak B. T. Findik D. Doruker P. (2018). RESPEC Incorporates Residue Specificity and the Ligand Effect into the Elastic Network Model. J. Phys. Chem. B 122, 5347–5355. 10.1021/acs.jpcb.7b10325 29268615

[B43] Kaynak B. T. Zhang S. Bahar I. Doruker P. (2021). ClustENMD: Efficient Sampling of Biomolecular Conformational Space at Atomic Resolution. Bioinformatics 37, 3956–3958. 10.1093/bioinformatics/btab496 PMC8570821 34240100

[B44] Koehl P. Orland H. Delarue M. (2021). Parameterizing Elastic Network Models to Capture the Dynamics of Proteins. J. Comput. Chem. 42, 1643–1661. 10.1002/jcc.26701 34117647

[B45] Krieger J. M. Doruker P. Scott A. L. Perahia D. Bahar I. (2020). Towards Gaining Sight of Multiscale Events: Utilizing Network Models and normal Modes in Hybrid Methods. Curr. Opin. Struct. Biol. 64, 34–41. 10.1016/j.sbi.2020.05.013 32622329 PMC7666066

[B46] Kurkcuoglu O. Jernigan R. L. Doruker P. (2006). Loop Motions of Triosephosphate Isomerase Observed with Elastic Networks. Biochemistry 45, 1173–1182. 10.1021/bi0518085 16430213 PMC2556966

[B47] Kurkcuoglu Z. Bahar I. Doruker P. (2016). ClustENM: ENM-Based Sampling of Essential Conformational Space at Full Atomic Resolution. J. Chem. Theor. Comput. 12, 4549–4562. 10.1021/acs.jctc.6b00319 PMC5088496 27494296

[B48] Kurkcuoglu Z. Bonvin A. M. J. J. (2020). Pre‐ and post‐docking Sampling of Conformational Changes Using ClustENM and HADDOCK for Protein‐protein and protein‐DNA Systems. Proteins 88, 292–306. 10.1002/prot.25802 31441121 PMC6973081

[B49] Kurkcuoglu Z. Doruker P. (2016). Ligand Docking to Intermediate and Close-To-Bound Conformers Generated by an Elastic Network Model Based Algorithm for Highly Flexible Proteins. PLoS One 11, e0158063. 10.1371/journal.pone.0158063 27348230 PMC4922591

[B50] Kurkcuoglu Z. Doruker P. (2013). Substrate Effect on Catalytic Loop and Global Dynamics of Triosephosphate Isomerase. Entropy 15, 1085–1099. 10.3390/e15031085

[B51] Kurkcuoglu Z. Findik D. Akten E. D. Doruker P. (2015). How an Inhibitor Bound to Subunit Interface Alters Triosephosphate Isomerase Dynamics. Biophysical J. 109, 1169–1178. 10.1016/j.bpj.2015.06.031 PMC4576151 26190635

[B52] Lindorff-Larsen K. Piana S. Palmo K. Maragakis P. Klepeis J. L. Dror R. O. (2010). Improved Side-Chain Torsion Potentials for the Amber ff99SB Protein Force Field. Proteins 78, 1950–1958. 10.1002/prot.22711 20408171 PMC2970904

[B53] Maldonado E. Soriano-García M. Moreno A. Cabrera N. Garza-Ramos G. Tuena de Gómez-Puyou M. (1998). Differences in the Intersubunit Contacts in Triosephosphate Isomerase from Two Closely Related Pathogenic Trypanosomes. J. Mol. Biol. 283, 193–203. 10.1006/jmbi.1998.2094 9761683

[B54] Martin P. Vickrey J. F. Proteasa G. Jimenez Y. L. Wawrzak Z. Winters M. A. (2005). "Wide-Open" 1.3 Å Structure of a Multidrug-Resistant HIV-1 Protease as a Drug Target. Structure 13, 1887–1895. 10.1016/j.str.2005.11.005 16338417

[B55] Miyashita O. Tama F. (2018). Hybrid Methods for Macromolecular Modeling by Molecular Mechanics Simulations with Experimental Data. Adv. Exp. Med. Biol. 1105, 199–217. 10.1007/978-981-13-2200-6_13 30617831

[B56] Morningstar M. L. Roth T. Farnsworth D. W. Kroeger Smith M. Watson K. Buckheit R. W. (2007). Synthesis, Biological Activity, and crystal Structure of Potent Nonnucleoside Inhibitors of HIV-1 Reverse Transcriptase that Retain Activity against Mutant Forms of the Enzyme. J. Med. Chem. 50, 4003–4015. 10.1021/jm060103d 17663538 PMC3057568

[B57] Namasivayam V. Vanangamudi M. Kramer V. G. Kurup S. Zhan P. Liu X. (2019). The Journey of HIV-1 Non-nucleoside Reverse Transcriptase Inhibitors (NNRTIs) from Lab to Clinic. J. Med. Chem. 62, 4851–4883. 10.1021/acs.jmedchem.8b00843 30516990 PMC7049092

[B58] Onufriev A. Bashford D. Case D. A. (2004). Exploring Protein Native States and Large-Scale Conformational Changes with a Modified Generalized Born Model. Proteins 55, 383–394. 10.1002/prot.20033 15048829

[B59] Orellana L. (2019). Large-Scale Conformational Changes and Protein Function: Breaking the In Silico Barrier. Front. Mol. Biosci. 6, 117. 10.3389/fmolb.2019.00117 31750315 PMC6848229

[B60] Palese L. L. (2017). Conformations of the HIV-1 Protease: A crystal Structure Data Set Analysis. Biochim. Biophys. Acta (Bba) - Proteins Proteomics 1865, 1416–1422. 10.1016/j.bbapap.2017.08.009 28846854

[B61] Palmai Z. Chaloin L. Lionne C. Fidy J. Perahia D. Balog E. (2009). Substrate Binding Modifies the Hinge Bending Characteristics of Human 3-phosphoglycerate Kinase: a Molecular Dynamics Study. Proteins 77, 319–329. 10.1002/prot.22437 19422062

[B62] Palmai Z. Seifert C. Gräter F. Balog E. (2014). An Allosteric Signaling Pathway of Human 3-phosphoglycerate Kinase from Force Distribution Analysis. Plos Comput. Biol. 10, e1003444. 10.1371/journal.pcbi.1003444 24465199 PMC3900376

[B63] Phillips J. C. Hardy D. J. Maia J. D. C. Stone J. E. Ribeiro J. V. Bernardi R. C. (2020). Scalable Molecular Dynamics on CPU and GPU Architectures with NAMD. J. Chem. Phys. 153, 044130. 10.1063/5.0014475 32752662 PMC7395834

[B64] Resende-Lara P. T. Perahia D. Scott A. L. Braz A. S. K. (2020). Unveiling Functional Motions Based on point Mutations in Biased Signaling Systems: A normal Mode Study on Nerve Growth Factor Bound to TrkA. PLoS One 15, e0231542. 10.1371/journal.pone.0231542 32497034 PMC7272051

[B65] Schlitter J. Engels M. Krüger P. (1994). Targeted Molecular Dynamics: a New Approach for Searching Pathways of Conformational Transitions. J. Mol. Graphics 12, 84–89. 10.1016/0263-7855(94)80072-3 7918256

[B66] Scott W. R. P. Schiffer C. A. (2000). Curling of Flap Tips in HIV-1 Protease as a Mechanism for Substrate Entry and Tolerance of Drug Resistance. Structure 8, 1259–1265. 10.1016/s0969-2126(00)00537-2 11188690

[B67] Sluis-Cremer N. Temiz N. Bahar I. (2004). Conformational Changes in HIV-1 Reverse Transcriptase Induced by Nonnucleoside Reverse Transcriptase Inhibitor Binding. Curr. HIV Res. 2, 323–332. 10.2174/1570162043351093 15544453 PMC1298242

[B68] Swift R. V. McCammon J. A. (2008). Catalytically Requisite Conformational Dynamics in the mRNA-Capping Enzyme Probed by Targeted Molecular Dynamics. Biochemistry 47, 4102–4111. 10.1021/bi8000209 18330997

[B69] Thirumalai D. Hyeon C. Zhuravlev P. I. Lorimer G. H. (2019). Symmetry, Rigidity, and Allosteric Signaling: From Monomeric Proteins to Molecular Machines. Chem. Rev. 119, 6788–6821. 10.1021/acs.chemrev.8b00760 31017391

[B70] Tu X. Das K. Han Q. Bauman J. D. Clark A. D. Hou X. (2010). Structural Basis of HIV-1 Resistance to AZT by Excision. Nat. Struct. Mol. Biol. 17, 1202–1209. 10.1038/nsmb.1908 20852643 PMC2987654

[B71] Waskom M. (2021). Seaborn: Statistical Data Visualization. Joss 6, 3021. 10.21105/joss.03021

[B72] Williams J. C. McDermott A. E. (1995). Dynamics of the Flexible Loop of Triose-Phosphate Isomerase: The Loop Motion Is Not Ligand Gated. Biochemistry 34, 8309–8319. 10.1021/bi00026a012 7599123

[B73] Wingert B. Krieger J. Li H. Bahar I. (2021). Adaptability and Specificity: How Do Proteins Balance Opposing Needs to Achieve Function? Curr. Opin. Struct. Biol. 67, 25–32. 10.1016/j.sbi.2020.08.009 33053463 PMC8036234

[B74] Yamazaki T. Hinck A. P. Wang Y.-X. Nicholson L. K. Torchia D. A. Wingfield P. (1996). Three-dimensional Solution Structure of the HIV-1 Protease Complexed with DMP323, a Novel Cyclic Urea-type Inhibitor, Determined by Nuclear Magnetic Resonance Spectroscopy. Protein Sci. 5, 495–506. 10.1002/pro.5560050311 8868486 PMC2143364

[B75] Yang L.-W. Bahar I. (2005). Coupling between Catalytic Site and Collective Dynamics: a Requirement for Mechanochemical Activity of Enzymes. Structure 13, 893–904. 10.1016/j.str.2005.03.015 15939021 PMC1489920

[B76] Yang L. Song G. Carriquiry A. Jernigan R. L. (2008). Close Correspondence between the Motions from Principal Component Analysis of Multiple HIV-1 Protease Structures and Elastic Network Modes. Structure 16, 321–330. 10.1016/j.str.2007.12.011 18275822 PMC2350220

[B77] Yang L. Song G. Jernigan R. L. (2009). Protein Elastic Network Models and the Ranges of Cooperativity. Proc. Natl. Acad. Sci. 106, 12347–12352. 10.1073/pnas.0902159106 19617554 PMC2718344

[B78] Yon J. M. Perahia D. Ghélis C. (1998). Conformational Dynamics and Enzyme Activity. Biochimie 80, 33–42. 10.1016/s0300-9084(98)80054-0 9587660

[B79] Zerrad L. Merli A. Schröder G. F. Varga A. Gráczer É. Pernot P. (2011). A spring-loaded Release Mechanism Regulates Domain Movement and Catalysis in Phosphoglycerate Kinase. J. Biol. Chem. 286, 14040–14048. 10.1074/jbc.m110.206813 21349853 PMC3077604

[B80] Zhang S. Krieger J. M. Zhang Y. Kaya C. Kaynak B. Mikulska-Ruminska K. (2021). ProDy 2.0: Increased Scale and Scope after 10 Years of Protein Dynamics Modelling with Python. Bioinformatics 37, 3657. 10.1093/bioinformatics/btab187/6211036 PMC8545336 33822884

[B81] Zhang S. Li H. Krieger J. M. Bahar I. (2019). Shared Signature Dynamics Tempered by Local Fluctuations Enables Fold Adaptability and Specificity. Mol. Biol. Evol. 36, 2053–2068. 10.1093/molbev/msz102 31028708 PMC6736388

[B82] Zhang Y. Doruker P. Kaynak B. Zhang S. Krieger J. Li H. (2020). Intrinsic Dynamics Is Evolutionarily Optimized to Enable Allosteric Behavior. Curr. Opin. Struct. Biol. 62, 14–21. 10.1016/j.sbi.2019.11.002 31785465 PMC7250723

