# Supplementary material for: Sampling of Protein Conformational Space Using Hybrid Simulations: A Critical Assessment of Recent Methods
Source: Front Mol Biosci. 2022 Feb 4;9:832847. doi: 10.3389/fmolb.2022.832847 (PMC8855042; doi:10.3389/fmolb.2022.832847)
Supplement: Supplementary file 5 [file DataSheet1.DOCX]

Supplementary Material

# Supplementary Figures and Tables

## Supplementary Figures


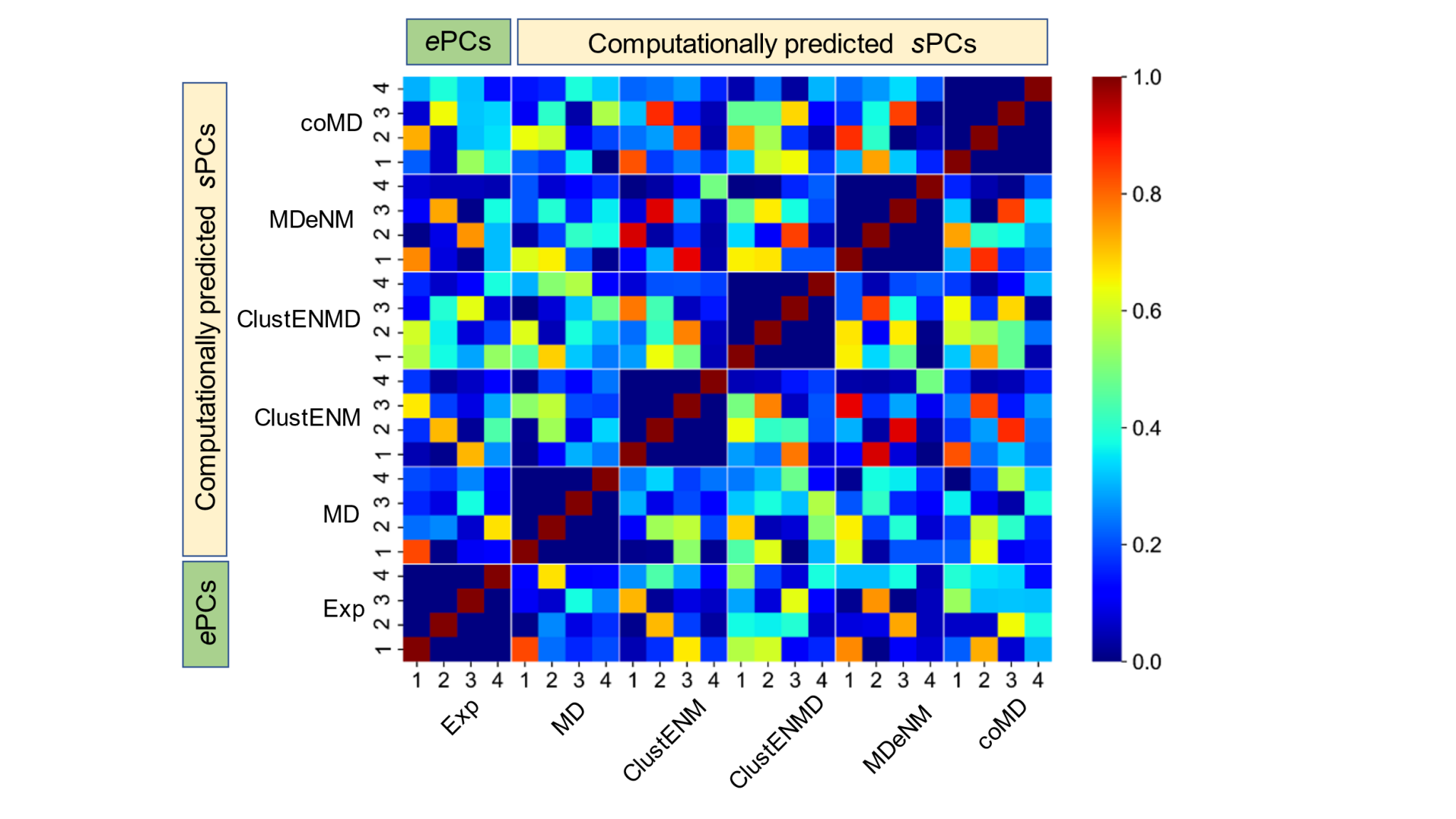


**Supplementary Figure 1.** **Overlap matrix for HIV-1 protease (PR).** Four-by-four submatices (a total of 6x6 submatrices separated by *thin white lines*) depict the correlation cosines between sets of PCs deduced from experiments, MD simulations, or hybrid methods, as indicated along the two axes. The entries are color-coded as indicated by the right bar.


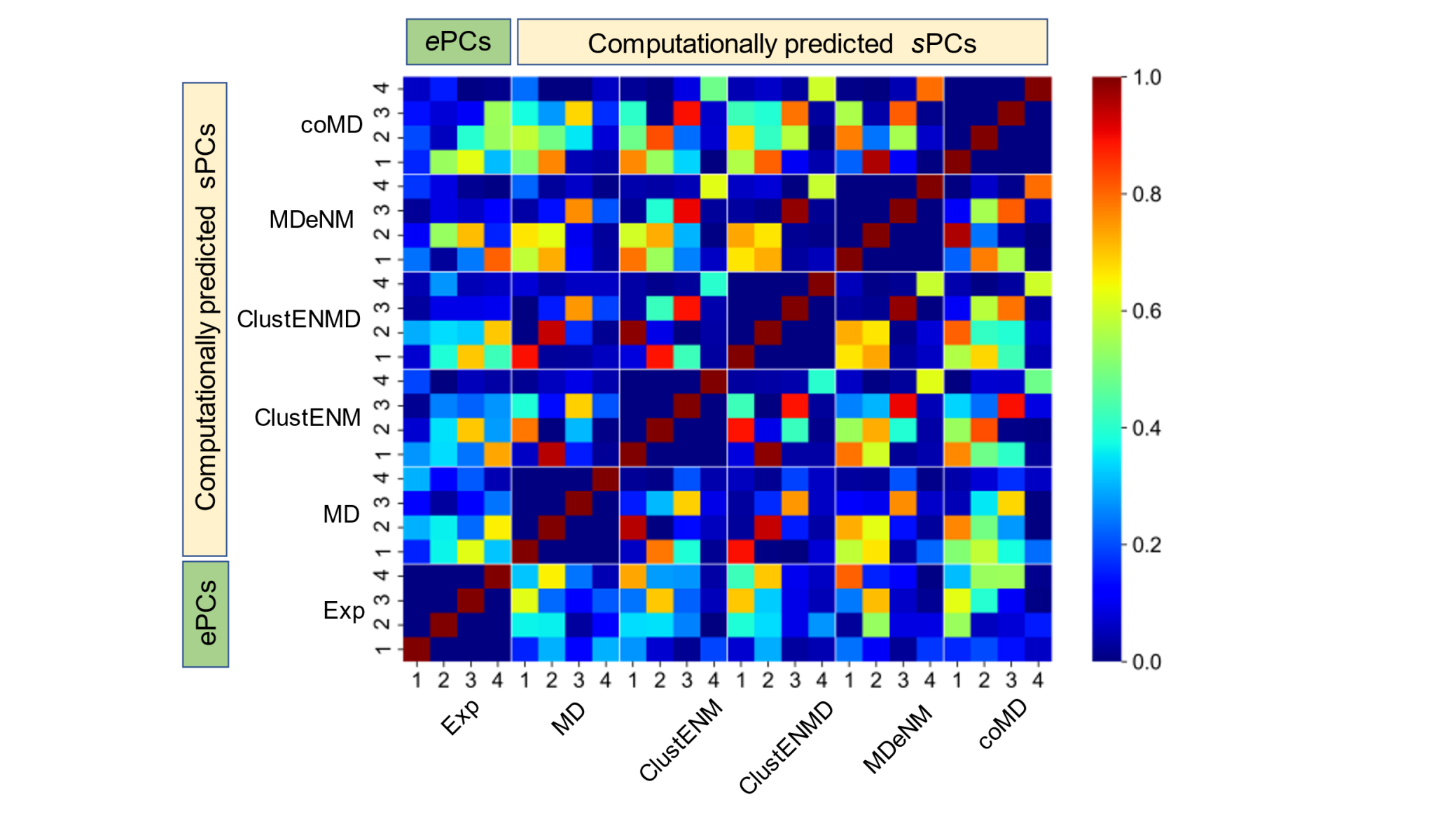


**Supplementary Figure 2.** **Overlap matrix for triosephosphate isomerase (TIM**). The matrix (or heat map) is in the same format as **Supplementary** **Figure 1**. See the caption for **Supplementary** **Figure 1.**


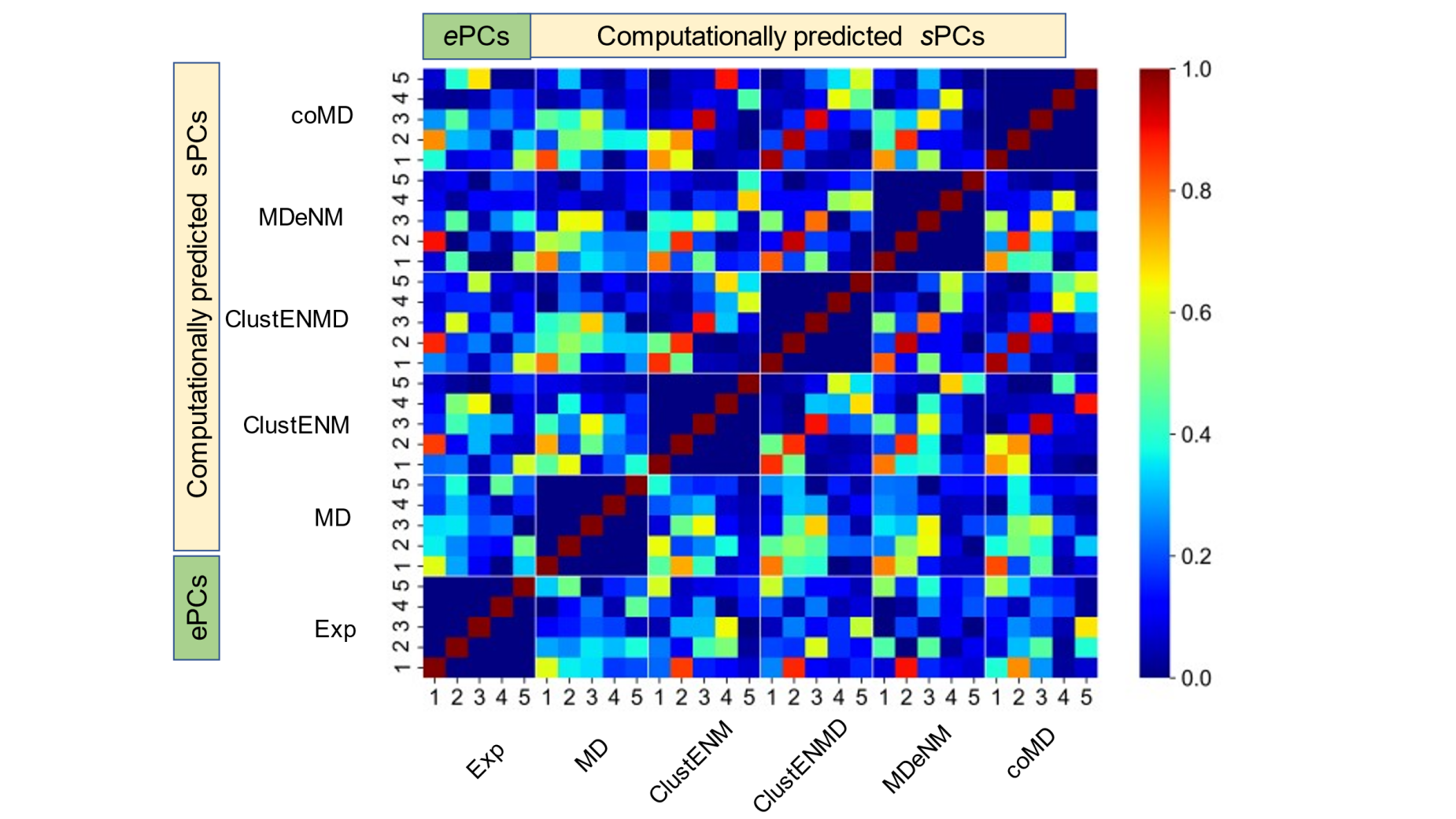


**Supplementary Figure 3.** **Overlap matrix for 3-phosphoglycerate kinase (PGK)**. The matrix (or heat map) is in the same format as **Supplementary** **Figure 1**. See the caption for **Supplementary** **Figure 1.**


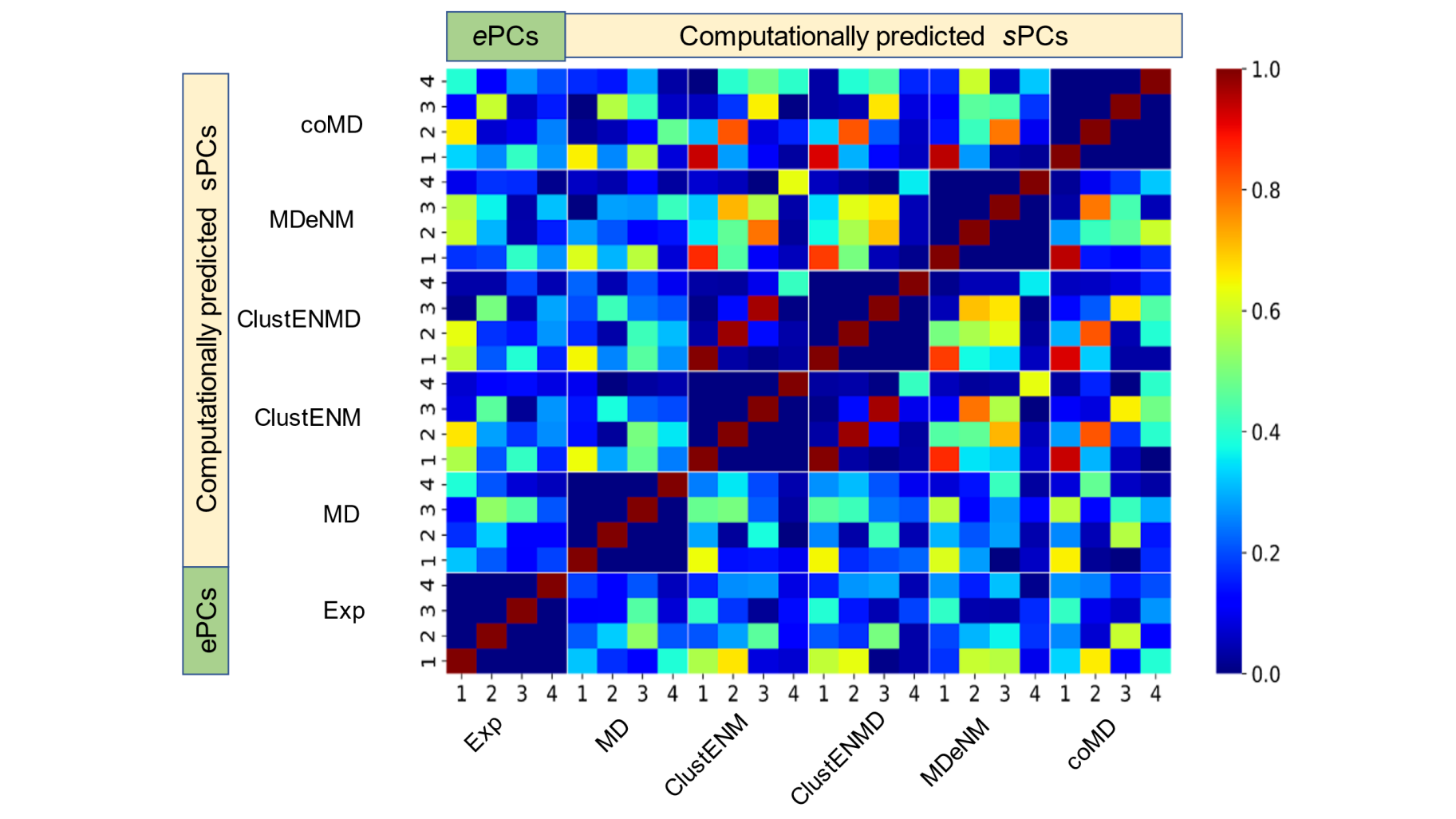


**Supplementary Figure 4.** **Overlap matrix for HIV-1 reverse transcriptase (RT)**. The matrix (or heat map) is in the same format as **Supplementary** **Figure 1**. See the caption for **Supplementary** **Figure 1.**


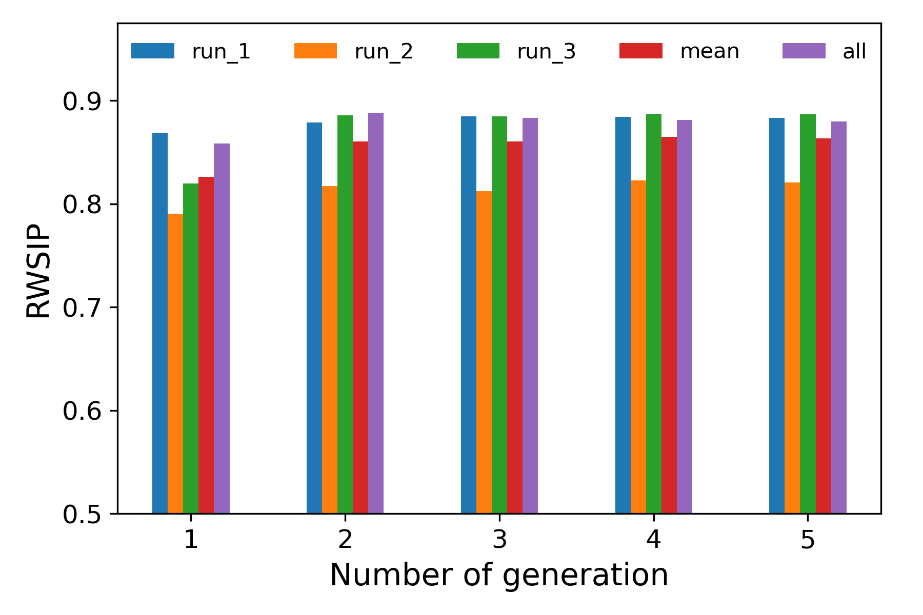

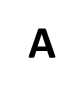

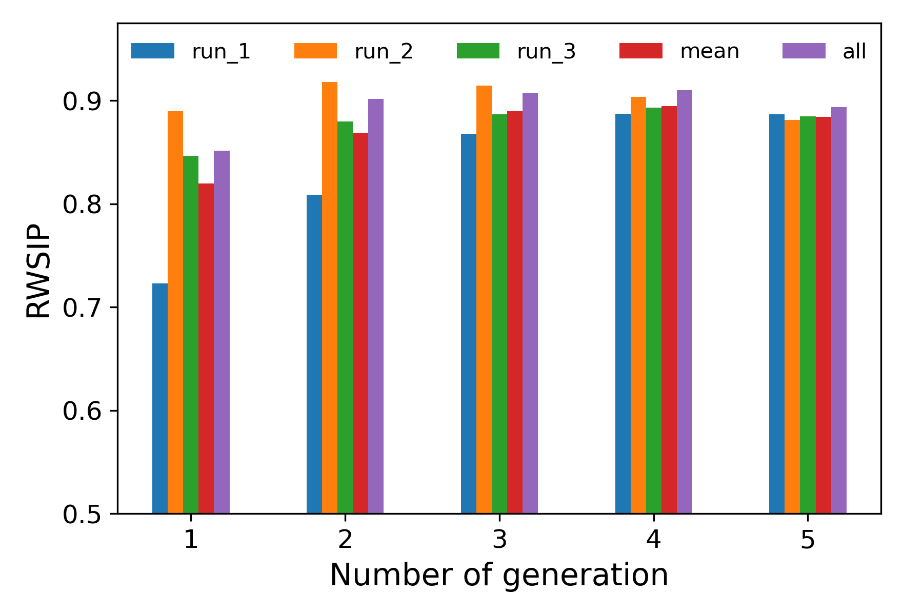

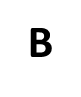


**Supplementary Figure 5. The progression of RWSIP values as a function of generations for (A) ClustENM and (B) ClustENMD.** RWSIP values are shown for each independent run. The *mean* bar stands for the RWSIP values averaged over three independent runs. The *all* bar indicates the analysis performed on the combined ensemble comprising all conformers coming from the three runs. Figures in both panels show that these two values converge after the second generation.


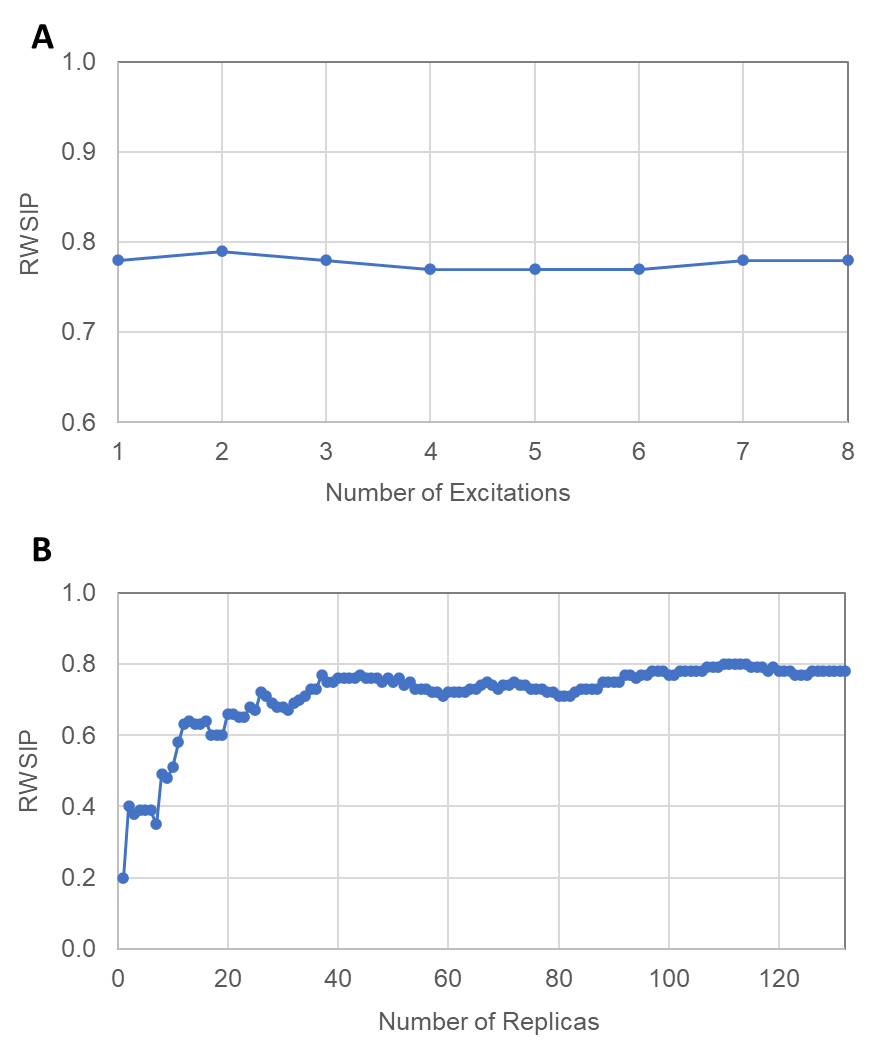


**Supplementary Figure 6. The progression of RWSIP values for MDeNM as a function of (A) number of excitations and (B) number of replicas.** Note the difference between the axis ranges, showing the higher values in **A**.

**
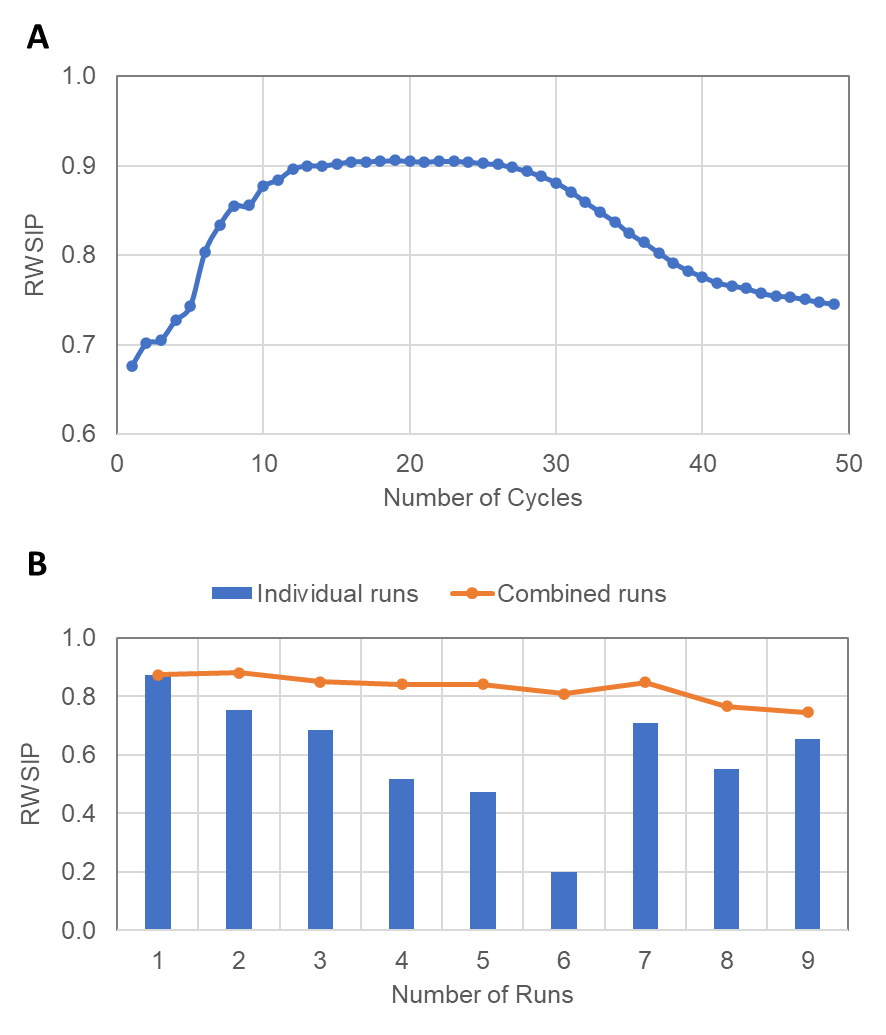
**

**Supplementary Figure 7. The progression of RWSIP values for coMD as a function of (A) number of cycles and (B) number of runs.** Note the difference between the axis ranges, showing the higher values in **A**.

## Supplementary Tables

**Supplementary Table 1. Comparison of computational efficiency**

| Method | Average run time (min) | | | | Features |
| --- | --- | --- | --- | --- | --- |
|  | PR | PGK | TIM | RT |  |
| ClustENM*^a^* | 4.7 | 6.9 | 8.0 | 19.6 | Full automation,  *ProDy* platform |
| ClustENMD*^a^* | 8.3 | 10.9 | 13.0 | 27.2 | Full automation,  *ProDy* platform |

*^a^ Average run time over 3 independent runs, each producing about 301 conformers. All runs were performed on a single GPU platform with NVIDIA GeForce RTX 2080 Ti graphics card. CPUs may vary from run to run.*
